# Dissociation between red and white stimulus perception: A perimetric quantification of protanopic color vision deficiencies

**DOI:** 10.1371/journal.pone.0260362

**Published:** 2021-12-20

**Authors:** Denise Wetzel, Judith Ungewiss, Michael Wörner, Helmut Wilhelm, Ulrich Schiefer

**Affiliations:** 1 Study course Ophthalmic Optics/Optometry, Aalen University of Applied Sciences, Aalen, Germany; 2 Competence Center Vision Research / Study course Ophthalmic Optics/Optometry, Aalen University of Applied Sciences, Aalen, Germany; 3 Carl Zeiss Vision International GmbH, Aalen, Germany; 4 Department of Ophthalmology, Tübingen University, Tübingen, Germany; 5 Blickshift GmbH, Stuttgart, Germany; L V Prasad Eye Institute, INDIA

## Abstract

**Significance:**

Horizontal visual field extension was assessed for red and white stimuli in subjects with protanopia using semi-automated kinetic perimetry. In contrast to a conventional anomaloscope, the “red/white dissociation ratio” (RWR) allows to describe protanopia numerically. For the majority of subjects with protanopia a restriction for faint red stimuli was found.

**Purpose:**

Comparing the horizontal visual field extensions for red and white stimuli in subjects with protanopia and those with normal trichromacy and assessing the related intra-subject intra-session repeatability.

**Methods:**

The subjects were divided into groups with protanopia and with normal trichromacy, based on color vision testing (HMC anomaloscope, Oculus, Wetzlar/FRG). Two stimulus characteristics, III4e and III1e, according to the Goldmann-classification, were presented with semi-automated kinetic perimetry (Octopus 900 perimeter, Haag-Streit, Köniz/CH). They moved along the horizontal meridian, with an angular velocity of 3°/s towards the visual field center, starting from either the temporal or nasal periphery. If necessary, a 20° nasal fixation point offset was chosen to capture the temporal periphery of the visual field. For each condition the red/white dissociation ratio (RWR); *Pat Appl*. *DPMA DRN 43200082D)* between the extent of the isopter for *red* (RG610, Schott, Mainz/ FRG) and *white* stimuli along the horizontal meridian was determined.

**Results:**

All data are listed as median/interquartile range: Five males with protanopia (age 22.1/4.5 years) and six males with normal trichromacy (control group, age 30.5/15.2 years) were enrolled. The RWR is listed for the right eye, as no clinically relevant difference between right and left eye occurred. Protanopes’ RWR for mark III4e (in brackets: control group) was 0.941/0.013 (0.977/0.019) and for mark III1e 0.496/0.062 (0.805/0.051), respectively.

**Conclusions:**

In this exploratory “proof-of-concept study” red/white dissociation ratio perimetry is introduced as a novel technique aiming at assessing and quantifying the severity of protanopia. Further effort is needed to understand the magnitude of the observed red-/white dissociation and to extend this methodology to a wider age range of the sample and to anomalous trichromacies (protanomalia) with varying magnitude.

## Introduction

The ability of distinguishing colors is beneficial for many activities of daily living, including job performance [1]. Protanopia is a congenital color vision deficiency and is associated with a *“disturbance in the X-linked opsin gene array”* [2]. Because this is an x-linked recessive condition women are much less frequently affected than men (0.02 vs. 1.01%) [2,3]. This genetic modification results in a loss of light sensitivity on the long wavelength end of the visible spectrum [2,3]. This effect is attenuated by the comparatively broad overlap of the spectral sensitivity curves of medium-wavelength (M) and long-wavelength (L) cones with sensitivity maxima relatively close to each other [4]. Additionally, the (functional) absence of one receptor type causes problems in distinguishing red and green hues precisely. Polymorphism of e.g. (L) cones and inhomogeneous retinal distribution of various cone subtypes may lead to an intra-individual topography-/eccentricity-related variation of color sensitivity [4,5].

These conditions have an impact on many aspects of daily living: For a variety of driving, control and monitoring tasks, e.g. railway transportation [6], shipping [7], and air traffic [8], a correct and fast color discrimination is an indispensable precondition and therefore color vision testing of the employees within the scope of occupational health examinations is rated as essential [9]. These regulations have their roots in 1875, where a color vision deficiency was claimed to be the principal cause of a railway accident [10]. With regard to driving performance, many epidemiologic studies have been conducted to investigate whether protanopia is associated with a higher accident rate. However, the study findings differ widely between having no impact on driving performance to a significant increase of the accident rates of subjects with protanopia [11–13]. This circumstance has far-reaching implications for implementing regulations regarding (protan) color disturbances in occupational medicine and in the transportation sector.

A color vision deficiency can be detected with a series of color vision testing plates such as the pseudoisochromatic plates introduced by Ishihara or the Ichikawa SPP1 plate, Velhagen-Broschmann plates, color-sorting tests like Farnsworth Panel D15, Farnsworth Munsell 100 Hue test, Roth 28 Hue, or on the anomaloscope [14]. The latter instrument allows for the quantification of anomalous trichromacies (such as deuteranomaly or protanomaly) by using the Rayleigh equation and its related anomaly quotient (AQ)–with corresponding effects on the suitability assessment of the person concerned. According to the authors’ opinions, there are good reasons to assume such inter-individual differences of severity also for subjects with dichromatism, with implications on the color vision-related ability level, too. However, the anomaloscopic approach fails in case of dichromatism, as the resulting anomaly quotient varies between 0 and +∞ [14].

Standard white-on-white perimetry and the related extension of the visual field along the horizontal meridian is taken as an essential criterion for the assessment of driving ability. For instance, in Germany, *kinetic* perimetry, i.e., the presentation of *moving* stimuli is mandatory in the case of expert testimony. In color vision deficiencies specifically affecting the L-cones (as in protanomaly or protanopia), the spectral sensitivity is exclusively impaired in the long-wavelength color range. The additional use of long-wavelength (i.e. red) color stimuli is thus an intrinsic consequence. The comparison of the horizontal visual field extent, obtained with red and with white stimuli ensures a direct quantitative, easily comprehensible description of this functional impairment due to color vision impairment with the aid of a widely used examination method relevant to expert opinion. Perimetry along the *horizontal meridian* is considered the most useful method, since in many countries the *horizontal* visual field extent is used as a criterion for driving ability.

Therefore *the purpose* of this exploratory proof-of-concept study was to introduce the red white dissociation ratio (RWR) method [15] for assessing and numerically describing protanopia by relating the visual field extent obtained with *red* stimuli to that obtained with *white* stimuli using semi-automated kinetic perimetry (SKP) [16] and to assess its intra-subject, intra-session repeatability.

## Procedure

The subjects included in the study were male, healthy and at least 18 years old with a valid driver’s license. The following conditions led to an exclusion: Binocular best corrected visual acuity exceeding logMAR 0.1 –i.e. to less than 16/20 (SNELLEN fraction) corresponding to less than 0.8 (decimal fraction), respectively, spherical correction exceeding ±8 diopters, cylindrical correction exceeding ±2.5 diopters (in order to minimize the risk of refractive amblyopia and to avoid any relevant shift of the threshold location due to prismatic effects), strabismus causing double images, disease of the central nervous system or the optic nerve, relative afferent pupil defect (RAPD) exceeding 0.3 log units (in order to minimize the risk of a neuro-ophthalmologically relevant unilateral visual pathway lesion), abnormal anterior segments (slit lamp biomicroscopy), abnormal fundus exam (direct and indirect ophthalmoscopy with undilated pupils), any signs of current or status post intraocular inflammation, any medication that influences the reaction time or color vision and status post serious eye injury or intraocular surgery.

### Subjects and methods

This research was reviewed by an independent ethical review board (ethics committee of the State Medical Association Baden-Württemberg, internal reference no.: F-2019-074) and conforms with the principles and applicable guidelines for the protection of human subjects in biomedical research. A clinical trial registration and approval was conducted (ClinicalTrials.gov identifier: NCT04060238). Written informed consent of each participant was obtained prior to the measurements. Each subject’s general and ophthalmological-optical history was noted in a medical record sheet (see S1 Table). All collected data were pseudonymized and treated confidentially.

The following examinations were performed in a standardized manner:

Testing of the ocular alignment and ocular motility (corneal reflex images and duction test), pupil reaction (swinging flashlight test), the stereoscopic vision (Lang stereo test (I), Lang-Stereotest AG, Küsnacht/CH), slit lamp biomicroscopy of the anterior segments (BQ 900, Haag-Streit Inc., Köniz/CH), direct ophthalmoscopy (Beta 2000, Heine Optotechnik Inc., Herrsching/FRG) and indirect binocular ophthalmoscopy (Omega 500, Heine Optotechnik Inc., Herrsching/FRG) with undilated pupils, each. The refractive power of the habitual glasses was measured with an automated lens meter (HLM-9000, Huvitz Inc., Hannover/FRG) and subjective refraction was assessed using the phoropter (MeDOP MP-500, Pacific Optics Electronics Co., Ltd.; GuangDing/CHN). For assessment of (distant) visual acuity, optotypes (numbers) were presented in a distance of 5.4 m on a monitor (Visucat, IBK Systeme GmbH, Aalen/FRG) (see S2 Table).

Vision-related quality of life was assessed with the validated German version of the NEI-VFQ-25 questionnaire which includes aspects like general health or personal vision-related assessment in certain situations [17,18]. The achieved scores served for comparing the experiences of daily living between subject with protanopia and those with normal trichromacy.

The participants were divided into subjects with protanopia and those with normal trichromacy, based on the results of standardized color vision testing on the HMC anomaloscope (Oculus, Wetzlar/FRG) with automated neutral adaption periods of 3 seconds each after a presentation interval of 3 seconds each. In all cases of normal or anomalous trichromacy the related matching ranges were assessed. Matching ranges between 34 and 46 units at a yellow brightness setting of 15±3 units were rated as normal trichromacy. The examination procedure was performed manually.

The perception thresholds for red and white stimuli were measured using the patented method of semi-automated kinetic perimetry (SKP) (Octopus 900, Co., Haag-Streit, Köniz/CH) [16] for testing and quantifying the novel red/white dissociation ratio (RWR). [9] Red (RG610, Schott Inc., Mainz/FRG) and white stimuli with the Goldmann characteristics III4e with the nominal values (25.7’, 320 cd/m^2^) and III1e with the nominal values (25.7’, 10 cd/m^2^) were presented with an angular velocity of 3°/s. The above luminance data refer to the conventional “white” marks. The red filter (SCHOTT RG610) installed by the company was selected to generate the red stimuli. Despite a lower/inferior L-cone isolation, the SCHOTT long pass filter RG610 was preferred over filters with a cut-off in the longer wavelength range (e.g. RG630 or RG662), as in the latter cases the luminance level and the dynamic range would be markedly reduced due to the high blue light component of the LEDs installed in the perimeter [19].

Spectroradiophotometric measurements (spectroradiometer CAS140 CT VIS/UV, Instrument Systems, Munich/FRG, measuring head TOP100, measuring aperture 0.25 mm, with the lens f = 60 mm, f/2.8; measurement software: SpecWin Pro 3.3, Instrument Systems, Munich/FRG) were taken in the center of the perimeter dome for the *white* stimuli (the luminance measurement results for *red* marks of otherwise identical GOLDMANN properties are shown in brackets). *III4e* (relevant for the expert opinion): stimulus luminance 306.9 cd/m^2^ minus the measured background luminance of 10 cd/m^2^; the resulting difference is 296.9 cd/m^2^ (stimulus luminance 104.3 cd/m^2^ minus background luminance, resulting in 94.3 cd/m^2^, with a related *attenuation factor of 3*.*1*, *corresponding to 5*.*0 dB*) or *III1e*: stimulus luminance 20.2 cd/m^2^ minus background luminance (10 cd/m^2^), with a resulting difference of 10.2 cd/m^2^ (stimulus luminance 12.8 cd/m^2^ minus background luminance, with a resulting difference of 2.8 cd/m^2^, with a related *attenuation factor of 3*.*6*, *corresponding to 5*.*6 dB*). (For further information, see S1 File).

The stimuli moved from the temporal or nasal periphery towards the visual field center. To assess the perception at the far temporal periphery, the–otherwise centrally located–fixation location (green fixation stimulus: visual angle approx. 0.5°, luminance 33.7 cd/m^2^; spectroradiophotometric measurement method, see above) was shifted by 20° nasally along the horizontal meridian and replaced by a fixation mark with comparable extent and luminance which was generated with a green (frequency-doubled NdYAG) laser pointer, attenuated by an appropriate welding glass. The shift of the fixation location was taken into account for further evaluation.

The eccentricity positions along the horizontal meridian, at which the participant pushed the response button (corresponding to the nasal and temporal visual field borders) were recorded and exported to an Excel work sheet. Each data set was corrected for the individual reaction time of the respective participant as well as for the fixation shift, if applied. In order to assess repeatability and intra-individual local scatter, each kinetic stimulus condition was presented seven times.

The local kinetic threshold was defined as the *median* eccentricity obtained by presenting each stimulus seven times along each vector. The individual scatter of the threshold location was specified by the related interquartile range (IQR, i.e. the 25^th^ and 75^th^ percentile). The horizontal extent of the related isopter is defined by the sum of the *nasal* and the related *temporal* eccentricity (in degrees). The RWR value is defined as the ratio between the horizontal extent, obtained with the *red* stimulus (numerator), and the horizontal extent, obtained with the corresponding *white* stimulus (denominator). This procedure was carried out for the median (50^th^ percentile) as well as for the 25^th^ and 75^th^ percentiles (, specifying the interquartile range = IQR) in identical manner.

In addition, all local kinetic threshold values were corrected for the *individual*, *stimulus-related reaction time* by shifting each threshold location backwards (i.e. in the opposite direction of the stimulus movement) for avoiding an artificial shrinkage of the visual field [20] This was achieved by presenting so-called “reaction time vectors” (heading towards the fixation point, three times for each condition, each with prior notice) *within the intact*, *central 20° visual field* (angular velocity of 3°/s and a length of about 5°). Thereby the time span between the onset of the stimulus presentation and the subject’s response was assessed and compensated [16,21]. All measurements were conducted for the right eye, the left eye and binocularly, respectively. Additionally, the individual reaction times were analyzed for each subject.

For calculating the red white dissociation ratio (RWR), the horizontal extent of the visual field (more exactly speaking: of the related isopter), obtained with the *red* stimulus, was divided by the extent obtained with the *white* stimulus of otherwise identical Goldmann characteristics. The RWR can take values between 1 (no restriction for red stimuli) to 0 (total loss for red stimuli) [15]. The RWR value is directly proportional to the (horizontal) visual field restriction for the red stimuli.

### Statistics

Due to the small sample size and in order to minimize the effects of any outliers, the data analysis was limited to descriptive, non-parametric (median, confidence interval) statistics: The listed RWR value is the median of all measured values and the box represents the interquartile range (IQR, i.e. the span between the 25^th^ and 75^th^ percentile). All presented data are listed as median/interquartile range. The statistics software JMP (Version: JMP 14 SW and JMP Pro 15; each: SAS Institute, Cary/USA) was used for comparison of the two subgroups (protanopia and normal trichromacy = control group) including the visualization of the results. The graphics were then edited with the open-source graphics software Inkscape (Version 0.92.2, Copyright 2003–2017 Inkscape Developers, GNU General Public License).

Sample size estimation for a subsequent study was performed with the statistics software G*Power (Version 3.1.9.6; Department of Psychology, University of Düsseldorf/FRG). The analysis for a two-tailed, nonparametric distribution was used and corrected with an asymptotic relative efficiency (ARE) of 0.955 [22], an alpha of *α* = 0.01* (*Bonferroni correction for multiple testing of 5 tests: comparison of right and left eye as well as intergroup comparison (protanopia vs. normal trichromacy) of the RWR, the reaction times and the NEI-VFQ questionnaire), an allocation ratio of 1 and a power of 0.8, which is routinely applied for standard clinical testing [23].

## Results

**Fig 1:**
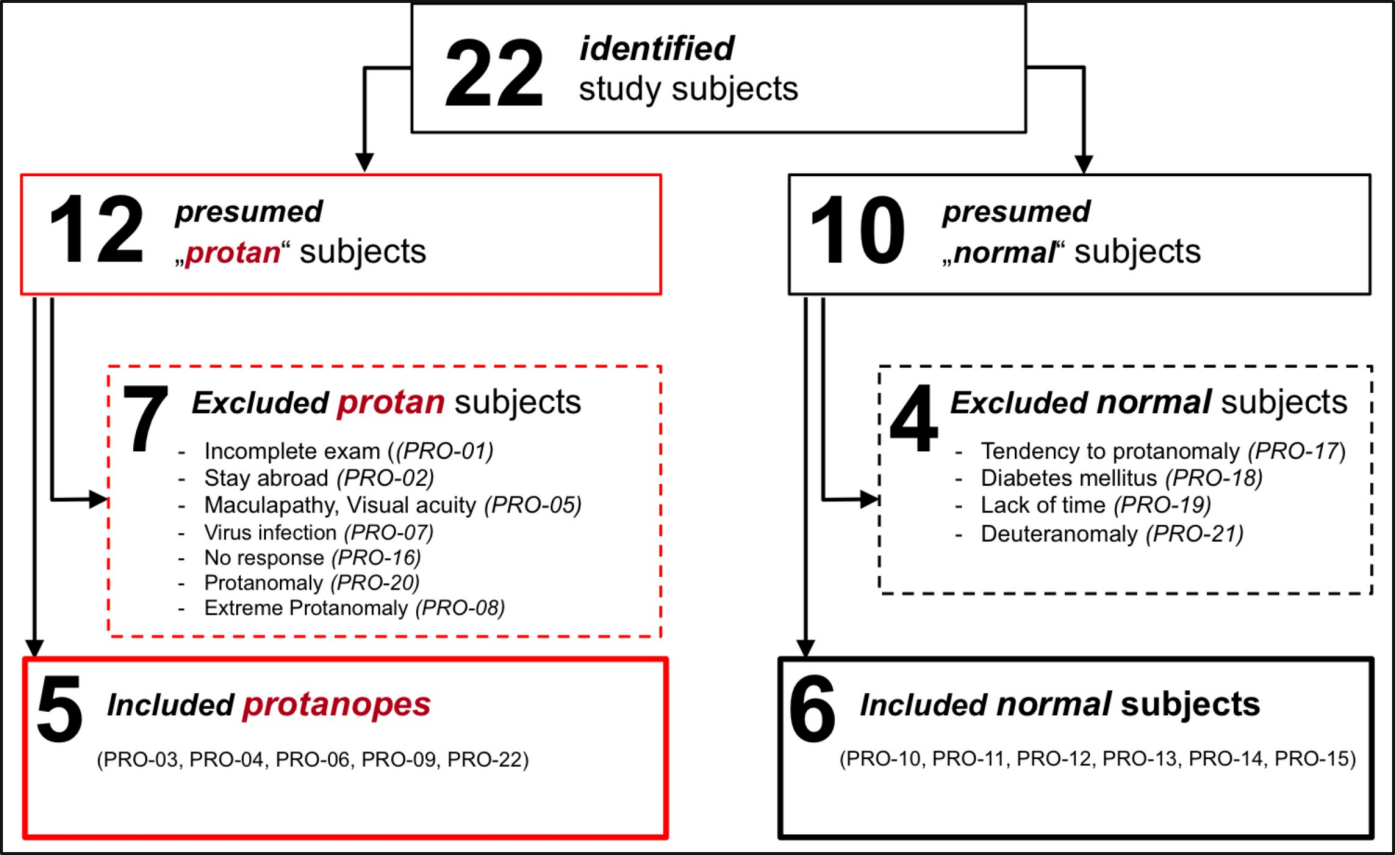
Inclusion/Exclusion process of the study participants. The flowchart illustrates the number of identified subjects at the beginning and the number of finally included subjects among the reasons for exclusion. The red boxes represent the subjects with protanopia; the black boxes represent the control group.

For recruitment, the Department of Ophthalmology at the Tübingen University as well as several ophthalmologists and eye clinics were contacted. Fig 1 illustrates the inclusion/exclusion process of the study: in total 22 male subjects were identified as potential participants, 12 presumed subjects with protanopia and 10 presumed subjects with normal trichromacy. After the ophthalmic examination 5 subjects with protanopia and 6 subjects with normal trichromacy could be finally included. The others had to be excluded due to eye diseases (maculopathy, cone dystrophy, viral infection), detection of (extreme) protanomaly or deuteranomaly or other reasons, listed in Fig 1. One subject with protanopia had to be excluded subsequently due to cone dystrophy and a related drop of visual acuity below the inclusion criterion. The median age/IQR for all subjects was 26.0/7.8 years. The subjects with protanopia were, on average, younger (22.1/4.45 years) than the subjects with normal trichromacy (30.5/15.18 years). Visual acuity (logMAR) of subjects with protanopia (values in brackets: spherical equivalent of their habitual correction) was -0.2/0.1 (+0.25/1.00 dpt) for the right eye and -0.2/0.1 (+0.25/1.13 dpt) for the left eye. In the control group visual acuity was -0.3/0.0 (-0.31/0.31 dpt) for the right eye and -0.3/0.1 (-0.56/0.59 dpt) for the left eye (for further detail, see S3 Table).

### Red/White ratio (RWR)

The single data points could not be discriminated in the original perimeter-generated visual field excerpt due to overlap (see S1 Fig). For this reason, the results were visualized with the statistics software JMP.

**Fig 2:**
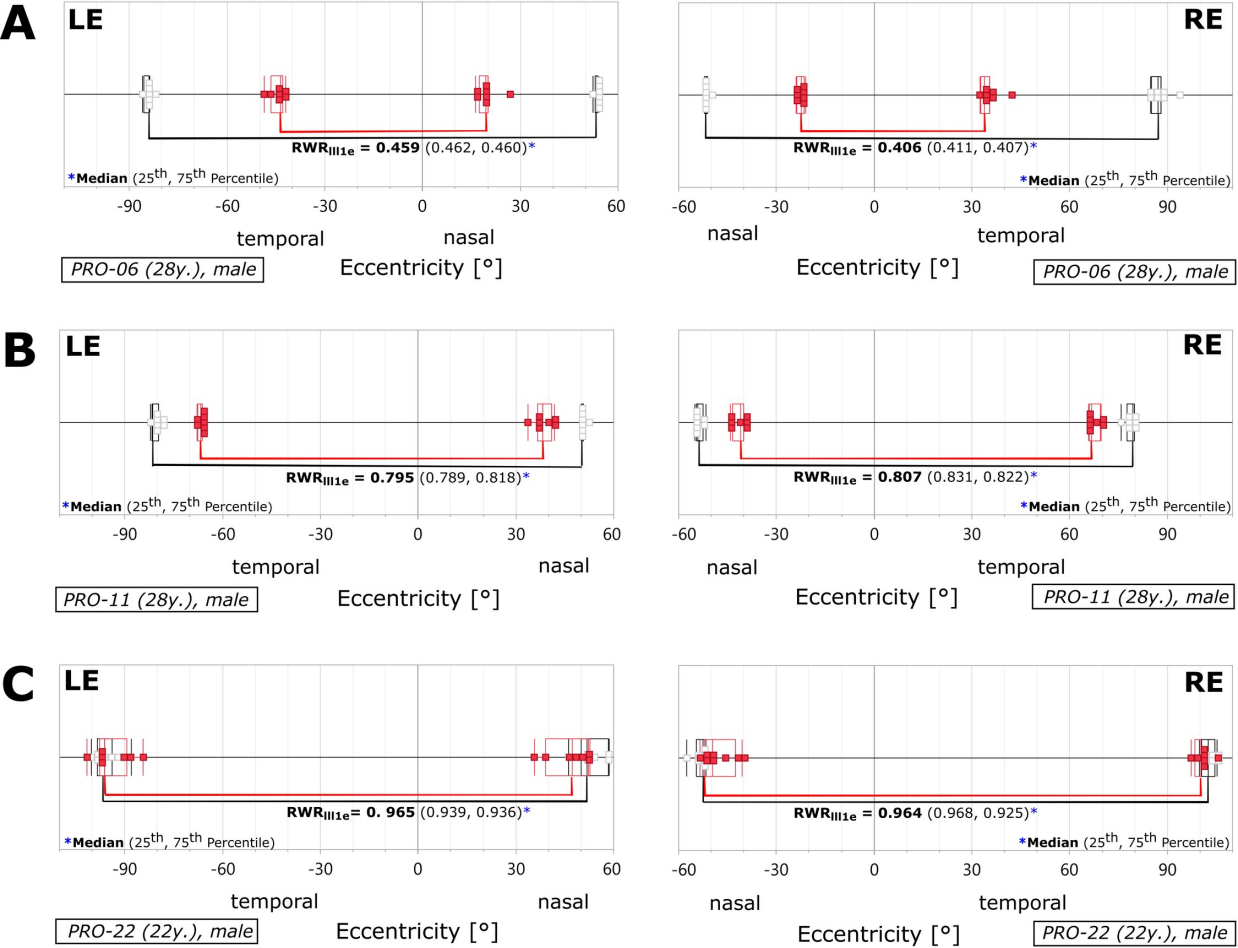
Graphical RWR analysis. Analysis of the subject with protanopia PRO-06 (A), the subject with normal trichromacy PRO-11 (B) and the subject with protanopia PRO-22 (C), separately for the right (RE) and left eye (LE). The data points are drawn onto a visual field map, showing the loci of the subjects´ stimulus detections. For analytical purposes, the points are superimposed to the boxplots. These boxplots are visualizing the statistical parameters *median* (middle line) and *interquartile range* (IQR: 25^th^ and 75^th^ quartile, lateral borders of the box). The individual thresholds of each condition were measured along the horizontal meridian (horizontal lines) by using the Goldmann stimulus characteristics III1e. The RWR was calculated by dividing the extent obtained for the *red* marks by the extent obtained for the *white* marks, respectively.

The inter-group comparison (control subjects vs. patients with protanopia) revealed an at most minimal RWR deviation in favor of the normal controls for *more intense* stimuli (III4e), indicating a ceiling effect. For this reason, the results obtained with the III4e mark could not be taken into account for the purpose of discrimination between subjects with protanopia and those with trichromacy. In contrast, there was a pronounced RWR decline in four out of five protanopic patients for *less intense* stimuli (III1e), indicating shrinkage of the horizontal visual field extent. This shrinkage is representatively shown for the subject with protanopia PRO-06 (A) in Fig 2. Subject PRO-22 (C) is the only tested subject with protanopia that does *not* follow this characteristic protanopic decline, despite unambiguous classification on the anomaloscope. As a reference for age, the results for the subject with normal trichromacy PRO-11 (B) are also included in Fig 2. In contrast to PRO-06 this subject showed just a minimal decrease of visual field extent for red stimuli (see S2 Fig for all self-created RWR-figures and see S4 Table for the complete data for the RWR median).

**Fig 3:**
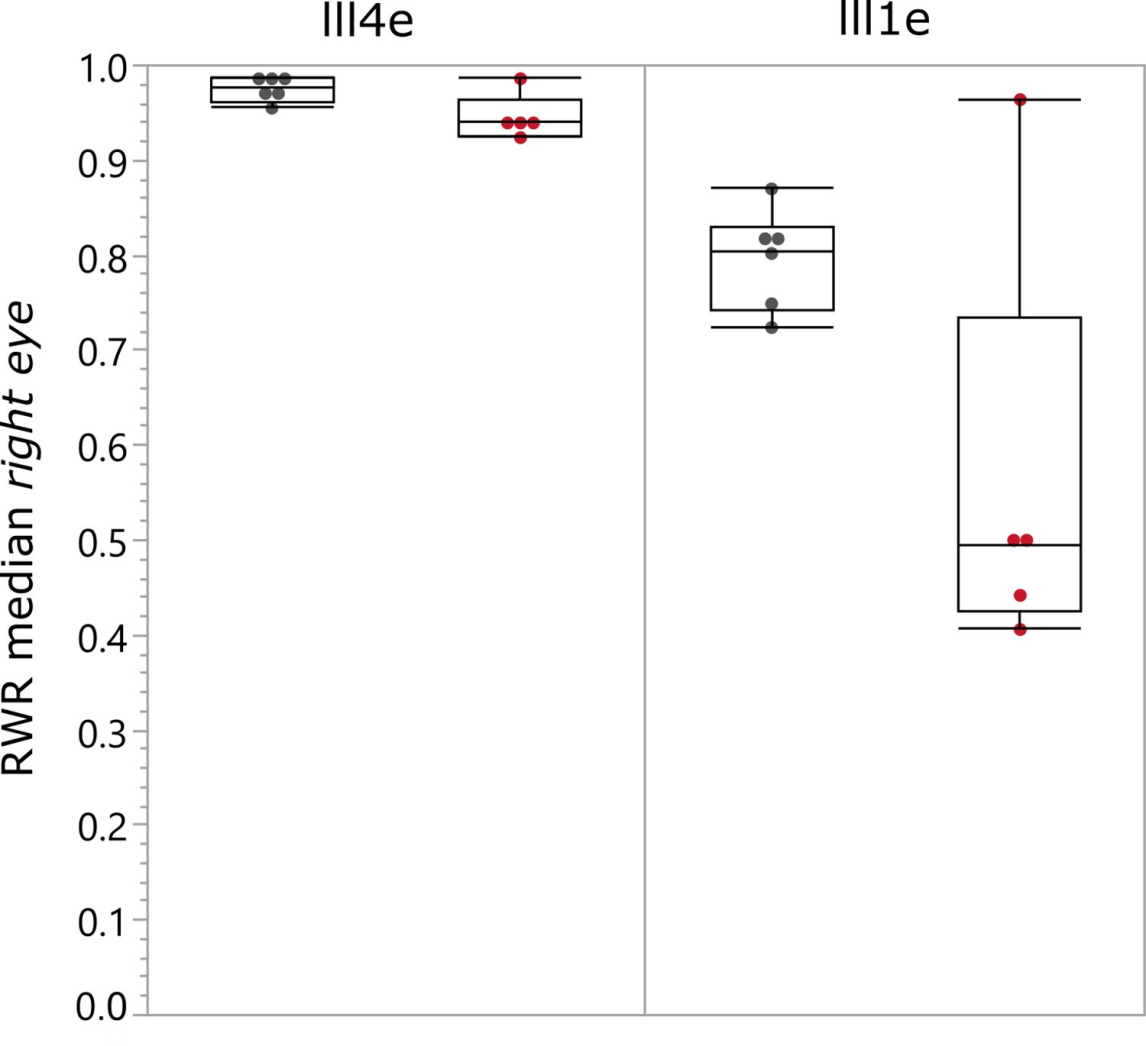
RWR medians for the right eyes of subjects with protanopia (red points, n = 5) and those with normal trichromacy (grey points, n = 6). The single data points are superimposed to the boxplots, which are visualizing the median (middle line) and interquartile range (IQR: 25^th^ and 75^th^ quartile, lateral borders of the box). The whiskers represent 1.5 times the interquartile range. The individual thresholds of each condition were measured along the horizontal meridian. The results are plotted separately for the two intensities III4e (left part) and III1e (right part).

Although a decrease of the horizontal extent for red, less intense stimuli in subjects with protanopia (except for PRO-22) could be observed (see Fig 2), the difference between the groups showed no clinically relevant difference, as can be seen in Fig 3. This is most probably caused by the deviating RWR result of the protanopic patient PRO-22 –despite a classical pathological anomaloscopic finding, indistinguishable from all other subjects with protanopia.

Since the right and left eye showed no clinically relevant difference, only the RWR results for the *right* eyes are shown in Fig 3.

### Reaction times

**Fig 4:**
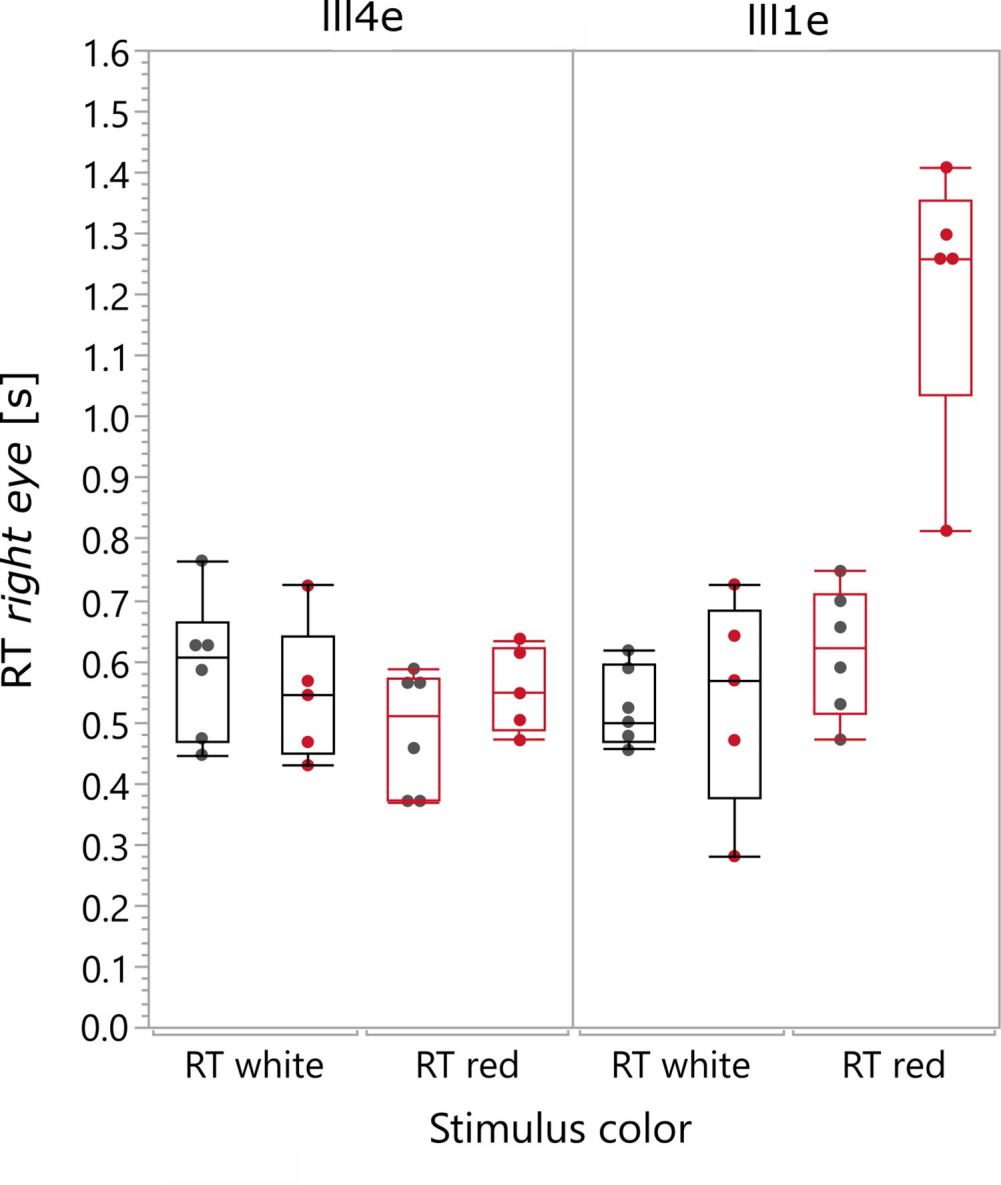
Graphical analysis of the reaction times RT [in seconds] for the subjects with protanopia (red points) and the control group with normal trichromacy (grey points). Each data point represents the calculated median out of the RT-measurement, repeated three times, for each condition. For description of the boxplot, see legend of Fig 3. The boxplots are shown separately for the intensities III4e and III1e and are additionally labeled for the stimulus color white (black boxes) and red (red boxes).

Fig 4 shows the reaction time (RT) assessment (in seconds) for each stimulus condition and intensity. Listed are only the RT values obtained for the right eyes, since no clinically relevant difference between right and left eyes occurred. Likewise to the RWR, only GOLDMANN stimuli III1e were statistically analyzed due to an insufficient differentiation ability of III4e mark, due to a ceiling effect: For the dim stimulus condition (III1e) the is a clear increase of the reaction time from 0.623/0.143 s (group median/group interquartile range = IQR, *red* stimulus, *normal trichromates*, RE) to 1.260/0.040 s (group median/group IQR, *red* stimulus, protanopia).

The inter-group comparison for the white stimuli showed no clinically relevance, whereas for the red stimuli a prolongation of the reaction time for subjects with protanopia could be observed (see S5 Table).

### NEI-VFQ questionnaire

The NEI-VFQ questionnaire provided no relevant information about a possible differentiation since the achieved scores of the subjects with protanopia lay within the same range as those of the subjects with normal trichromacy (data between the 25^th^ quartile and the 75^th^ quartile were overlapping). Therefore, no further statistical analysis was conducted (see S6 Table).

### Sample size estimation

Given the fact that sample sizes were small, a critical RWR effect size of 1.8 resulted as part of the sample size estimation [24]. With the current number of study participants only a statistical power of .34 could be reached. In order to achieve the usual clinically relevant power of .80 [23], the sample size will have to be increased to 20 participants– 10 participants in each group.

## Discussion

This study and its underlying sample has clearly proven the applicability of a conventional automated perimeter for assessing the red white dissociation ratio (RWR). The intra-session intra-subject repeatability of the results, obtained with RWR perimetry, is well comparable with that of conventional kinetic perimetry.

### Occupational and advisory relevance

As already mentioned, the ability of distinguishing colors is beneficial for many activities of daily living, including job performance [1]. Color vision deficiencies, especially protanopia is considered as a risk factor in road traffic and has led to a number of discussions, which resulted in filing color vision regulations [10]. Related studies provide different answers to the question, whether protanopic drivers cause significantly more car accidents than color normals: „Several studies found an impairment of driving ability as a consequence of color vision disorders, as patients reported to fail or have difficulties in seeing red signal or brake lights [25]. The recognition distance (visual range) for red was reduced [26] and increased reaction times and diminished correct identification for red are factors causing more accidents [12,27]”. In contrast, other studies did not find a significant over-representation of subjects with protanopia within the accident statistics and that they instinctively make use of cues like the traffic light order [11,28,29].

The DOG (German Ophthalmological Society) strongly recommends to exclude persons with an anomaly quotient lower than 0.5 –which would apply to all subjects with protanopia within this study–from holding a commercial driver’s licence [8]. International standards vary highly among countries, for example in Australia there is no requirement for color vision whereas in Colombia unrestricted color vision is mandatory [30].

### Reasons for the introduction of a perimetric approach

The idea of using colored stimuli for examining areas of spectral luminosity loss has been implemented for e.g. the red-perimetry for identification of protanopia-carriers [31]. This method is based on the knowledge that the visual field for the standard white on white perimetry is still normal, whereas changes for colored stimuli can already be detected [31]. The study made use of this effect by setting white and red visual field extents in relation to each other and creating the easily comprehensible “red/white dissociation ratio” (RWR) [15].

Kinetic perimetry was the method of choice for this study, since it “is more sensitive for detecting peripheral visual field defects” and “correlate[s] better with activities of daily living” [20]. For this reason, kinetic perimetry is used for a number of useful applications such as qualification for a driver’s license, the “assessment of visual performance and disability in relation to work and driving or assessment for other medical-legal purposes” [20,32].

The semi-automation of the kinetic method provides an even higher standardization with the implementation of a constant stimulus velocity, reduction of the examiner dependency to a minimum and, in contrast to a manual perimeter, the ability to measure the individual response time and remove false positive answers based on normal values [19,20]. The assessment of the reaction times requires particular attention since they can be influenced by age, stimulus luminance, eccentricity or, having the strongest impact, through intra-individual scatter [19]. In this study, age was not a factor, because all participants fell into the same age group. The study results indicated a potential increase of the reaction times in subjects with protanopia for the red Goldmann-mark III1e (even for a subject with protanopia without visual field constriction for less intense red stimuli).

### Disadvantages of conventional clinical color sense examinations

The examination on the anomaloscope requires a high amount of expertise–especially in case of anomalous trichromacy and its quantification, based on the assessment of the red/green matching results. In contrast, large part of the ophthalmologic assistant staff is able to conduct a conventional perimetric examination. This group can be familiarized with this perimetric method with comparably little effort. Furthermore, in contrast to an anomaloscope, a perimeter is available practically everywhere. Due to the similarity to conventional visual field measurements on the perimeter, most subjects are familiar with the test procedure.

The lack of evidence of significant differences between subjects with protanopia and those with normal trichromacy with respect of the NEI VFQ-25 scoring implies that the protanopic subjects usually do not experience a (subjective) restriction in everyday life with respect to color vision, which is probably attributable to individual experience and to the lack of direct inherent comparison with the color vision ability of subjects with normal trichromacy. There is only one query addressing color vision.

### Limitations of the current study

This study was an exploratory / proof-of-concept study, which was limited by the budget available and also by the threat of the emerging COVID-19 pandemic. The current sample size is too small to achieve a statistically significant power. This small sample size may be also the reason for (slight) differences with regard to the visual acuity level between the subjects with protanopia and the normal control group. Future studies should extend the age range of the sample in order to find out whether the RWR findings are influenced by age-related confounders, such as for instance media opacities or pupil size.

The measurements with the Goldmann mark III4e showed a ceiling effect for the RWR assessment in both groups, making the (supra-threshold) mark III4e ineffective with regards to the study purpose of discrimination between subjects with protanopia and the control group of those with normal trichromacy. The ceiling effect is due to the fact that for supra-liminal (white and red) stimuli *anatomical* outer boundaries (orbital bones, nose etc.) instead of retinal receptor-related properties act as limiting factors.

Further investigations for identifying the cause of the “atypical” behavior of subjects with protanopia like the subject PRO-22 should be considered. A possible explanation of this “mismatch” between anomaloscopic and perimetric examination could be due to the different retinal areas/eccentricities, addressed by the two methods: The anomaloscope examines the immediately (peri-)foveal 4°-diameter area, whereas the kinetic visual field testing addresses a considerably greater visual field eccentricity even when presenting less intense stimuli. [33,34] This shortcoming could be addressed by presenting *static* white and red stimuli at any desired location. In this case, the resulting *static RWR* is–according to the logarithmic scaling–the *difference* between the local differential luminance sensitivity values, obtained for *red* and for *white* static stimuli at the respective location.

In this study, only the spectral transmission of the red filter is shown, but not the actual power spectrum distribution that hits the eye of the person being examined. For any meaningful assessment, this would have to be considered at the retinal level, i.e. after passing through the refractive media of the eye. This is not readily possible, even in conventional perimetry nothing is known about the actual power spectrum at the retinal level.

Other studies confirmed similar theories of almost normal differential luminance sensitivity for red stimuli within the *peripheral* visual field in some subjects with protanopia [2,35]. A residual red-green discrimination, which can be explained by effects of rods in case of color matching with large fields is of no relevance for this study as comparatively small perimetric stimuli (≤ 26’) were used under photopic conditions (background luminance: 10 cd/m^2^) [36].

### Outlook

This constellation suggests examining the retinal distribution and the spectral sensitivity of single cone receptors using an adaptive optics Scanning Laser Ophthalmoscope (AOSLO) [37]. Early experiments detailing the cellular map of sensations originating from the mosaic of long, middle, and short wavelength-sensitive (L-, M-, S-) cones have led to intriguing hypotheses pertaining to the role of the cone mosaic and its ensuing pathways in mediating spatial and color vision [38–42]. Due to comparability reasons, differential luminance thresholds should be measured at predefined locations, matching those of the AOSLO. For this purpose, semi-automated kinetic perimetry should be supplemented by automated *static profile* perimetry using red and white stimuli, which is also covered by the above-mentioned patent [15].

## Conclusion

This study and its underlying sample has proven the applicability of a conventional automated perimeter for assessing the red/white dissociation ratio (RWR) in order to quantify the severity of protanopia. The intra-session intra-subject repeatability of the results, obtained with RWR perimetry, is well comparable with that of conventional kinetic perimetry.

Further effort is needed to understand the magnitude of the observed red-/white dissociation, e.g. by comparing to cell-targeted microstimulation [37,39] and to extend this methodology to a wider age range of the sample and to anomalous trichromacies (protanomalia) with varying magnitude.

## Supporting information

S1 Fig Original perimetric excerpts. The figure shows the original perimeter-generated file of the semi-automated kinetic perimetry results for the right eye (RE) and the left eye (LE) of two protanope subjects. The single points represent the local thresholds for white and red stimuli. The characteristics can be taken from the legend in the lower right corner. The reaction time measurements are located at an eccentricity of 20 degree nasal within the horizontal meridian. This figure description is also valid for all following figures of the original results illustration on the next pages.
(PDF)

S2 Fig RWR-figures. RWR analysis of the protanope subjects (A-E) and the normal trichromate PRO-11 (C), separately for the right (RE) and left eye (LE). The data points were drawn onto a visual field map, showing the locus of the subject’s stimuli detection. For analytical purposes the points are presented as boxplots, visualizing the statistical parameters *median* (middle line) and the *interquartile range* (IQR: 25^th^ and 75^th^ quartile, lateral borders of the box). By connecting the corresponding median thresholds of each condition the horizontal extent (horizontal lines) was obtained for the GOLDMANN stimulus characteristics III4e (thick lines) and III1e (thin lines). The RWR was calculated by dividing the III4e red extent by the III4e white extent, III1e respectively.
(PDF)

S1 Table Medical record sheet. Medical record sheet for the documentation of the subject’s general and ophthalmological optical history.
(PDF)

S2 Table Standard examination diagnosis sheet. Standard examination diagnosis sheet for the documentation of the subject’s performance or health findings.
(PDF)

S3 Table Subject’s visual acuity and habitual correction. Subject’s (PRO-ID as identification) habitual correction, objective/subjective refraction (sphere/cylindre/and visual acuity (VA_sc_ = visual acuity without correction; VA_cc_ = visual acuity with correction) listed for the right eye (RE), the left eye (LE) and binocularly (bin.).
(PDF)

S4 Table Complete data table of the RWR median. Red/white dissociation ratio results for every participant (PRO-ID as identification for the right eye (RE), left eye (LE) and both eyes (BE), seperately for the intensities III4e and III1e (shaded grey and bold letters).
(PDF)

S5 Table Complete data table of the reaction time assessment. Reaction times [sec] for all subjects (PRO-ID as identification) listed for the right eye (RE), left eye (LE) and both eyes (BE), separately for the intensities III4e and III1e (shaded grey and bold letters), for white and red respectively. The highlighted cells mark the false positive answers under the normal reference value of 180 ms (table extents over several pages).
(PDF)

S6 Table Table with the resulting scores of the NEI-VFQ questionnaire for each category. NEI-VFQ-scoring for every participant (PRO-ID as identification) represented by the different thematic parts of the questionnaire, highlighted is the color vision score for comparison purpose between protanopia and normal trichromasia.
(PDF)

S1 File Supplemental digital content. Detailed description of the experimental setup and the protocol of the spectroradiophotometric measurements.
(PDF)
