# Supplementary material for: Dissociation between red and white stimulus perception: A perimetric quantification of protanopic color vision deficiencies
Source: PLoS One. 2021 Dec 20;16(12):e0260362. doi: 10.1371/journal.pone.0260362 (PMC8687589; doi:10.1371/journal.pone.0260362)
Supplement: S1 Fig — The figure shows the original perimeter-generated file of the semi-automated kinetic perimetry results for the right eye (RE) and the left eye (LE) of two protanope subjects. The single points represent the local thresholds for white and red stimuli. The characteristics can be taken from the legend in the lower right corner. The reaction time measurements are located at an eccentricity of 20 degree nasal within the horizontal meridian. This figure description is also valid for all following figures of the original results illustration on the next pages. (PDF) [file pone.0260362.s001.pdf]

Supplemental Digital Content 5: Original perimetric excerpts

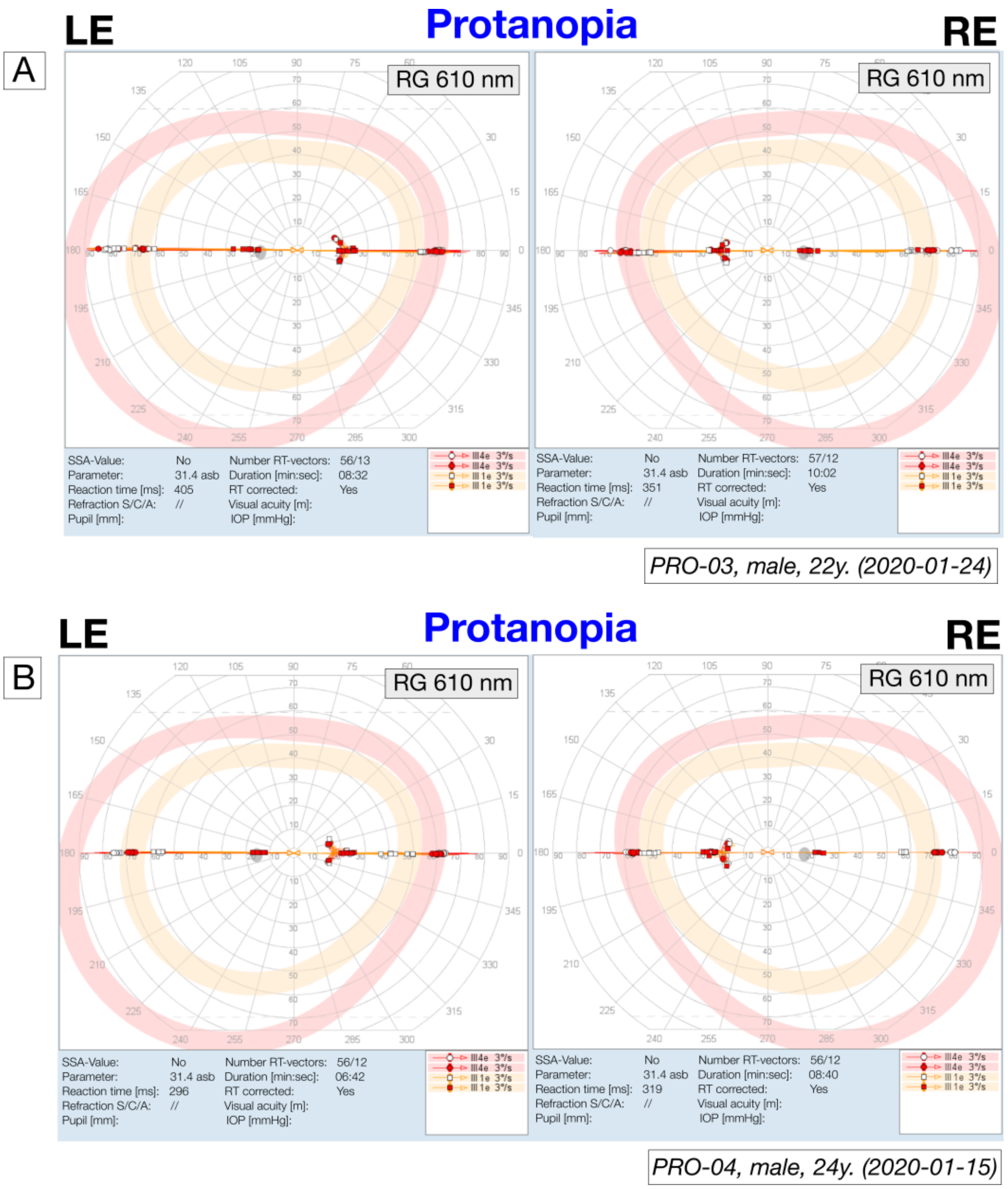

Figure: The figure shows the original perimeter-generated file of the semi-automated kinetic perimetry results for the right eye (RE) and the left eye (LE) of two protanope subjects. The single points represent the local thresholds for white and red stimuli. The characteristics can be taken from the legend in the lower right corner. The reaction time measurements are located at an eccentricity of 20 degree nasal within the horizontal meridian.

This figure description is also valid for all following figures of the original results illustration on the next pages

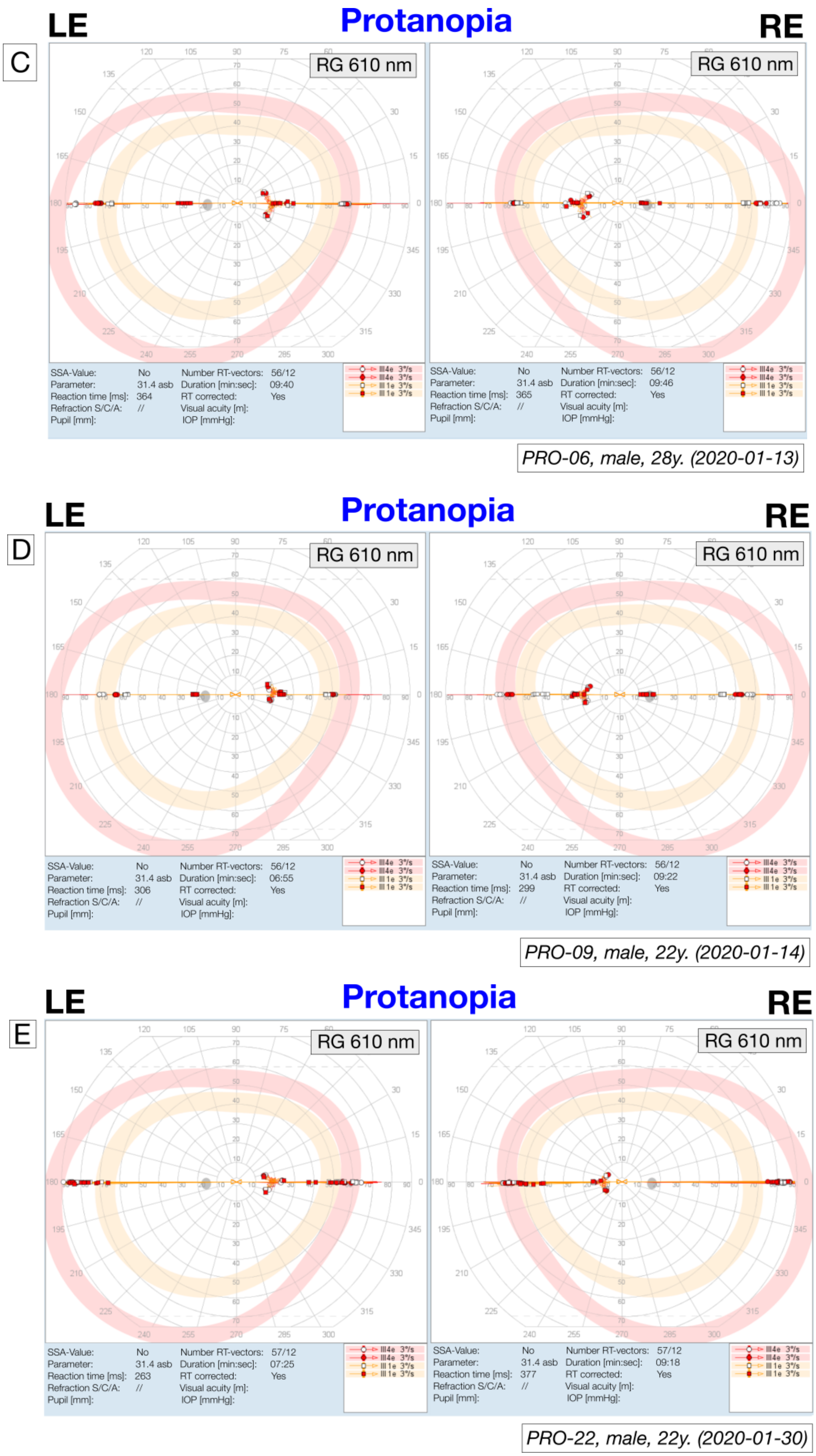
C
LE
Protanopia
RE
RG 610 nm
SSA-Value: No
Parameter: 31.4 asb
Reaction time [ms]: 364
Refraction S/C/A: //
Pupil [mm]:
Number RT-vectors: 56/12
Duration [min:sec]: 09:40
RT corrected: Yes
Visual acuity [m]:
IOP [mmHg]:
III4e 3°/s
III4e 3°/s
III 1e 3°/s
III 1e 3°/s
SSA-Value: No
Parameter: 31.4 asb
Reaction time [ms]: 365
Refraction S/C/A: //
Pupil [mm]:
Number RT-vectors: 56/12
Duration [min:sec]: 09:46
RT corrected: Yes
Visual acuity [m]:
IOP [mmHg]:
PRO-06, male, 28y. (2020-01-13)
D
LE
Protanopia
RE
RG 610 nm
SSA-Value: No
Parameter: 31.4 asb
Reaction time [ms]: 306
Refraction S/C/A: //
Pupil [mm]:
Number RT-vectors: 56/12
Duration [min:sec]: 06:55
RT corrected: Yes
Visual acuity [m]:
IOP [mmHg]:
SSA-Value: No
Parameter: 31.4 asb
Reaction time [ms]: 299
Refraction S/C/A: //
Pupil [mm]:
Number RT-vectors: 56/12
Duration [min:sec]: 09:22
RT corrected: Yes
Visual acuity [m]:
IOP [mmHg]:
PRO-09, male, 22y. (2020-01-14)
E
LE
Protanopia
RE
RG 610 nm
SSA-Value: No
Parameter: 31.4 asb
Reaction time [ms]: 263
Refraction S/C/A: //
Pupil [mm]:
Number RT-vectors: 57/12
Duration [min:sec]: 07:25
RT corrected: Yes
Visual acuity [m]:
IOP [mmHg]:
SSA-Value: No
Parameter: 31.4 asb
Reaction time [ms]: 377
Refraction S/C/A: //
Pupil [mm]:
Number RT-vectors: 57/12
Duration [min:sec]: 09:18
RT corrected: Yes
Visual acuity [m]:
IOP [mmHg]:
PRO-22, male, 22y. (2020-01-30)

F

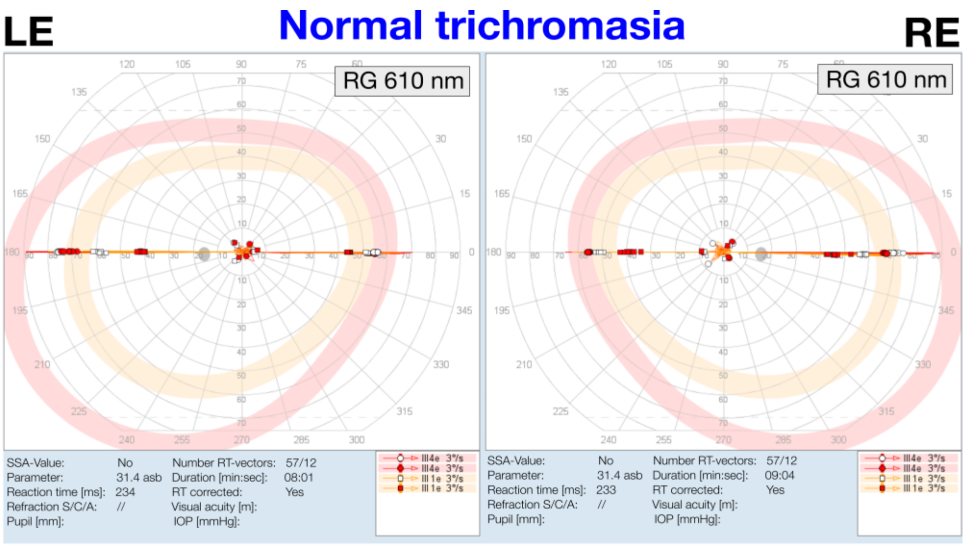

PRO-10, male, 38y. (2019-12-16)

G

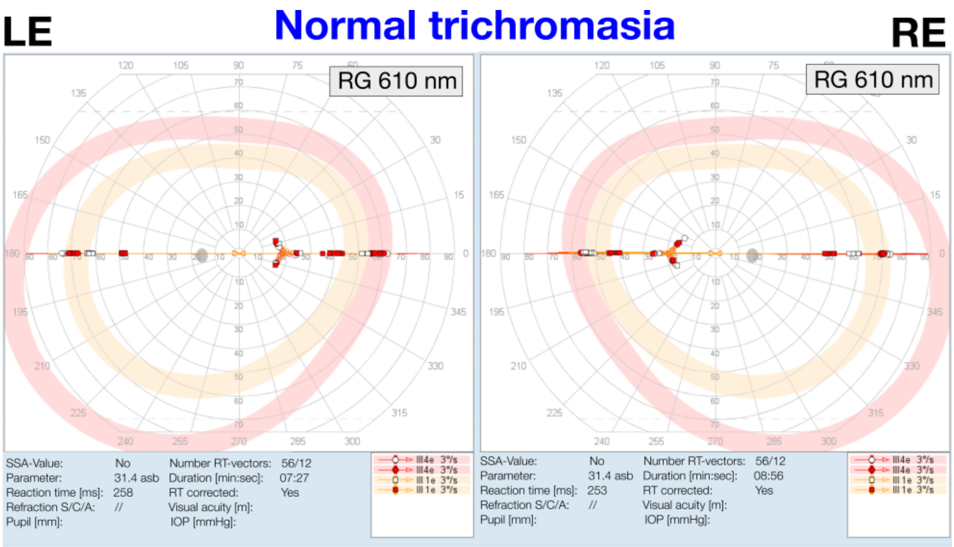

PRO-11, male, 28y. (2020-01-14)

H

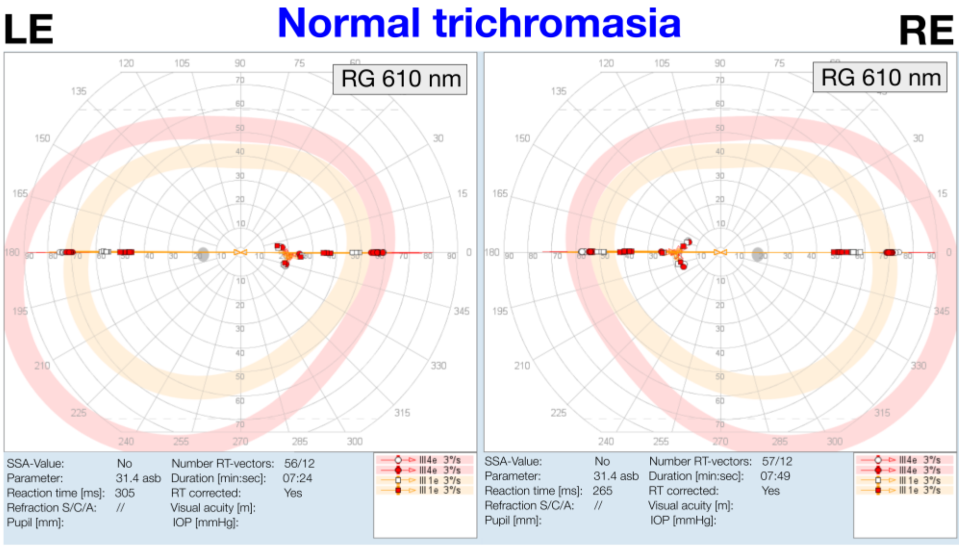

PRO-12, male, 33y. (2020-01-14)

LE **Normal trichromasia** RE

I

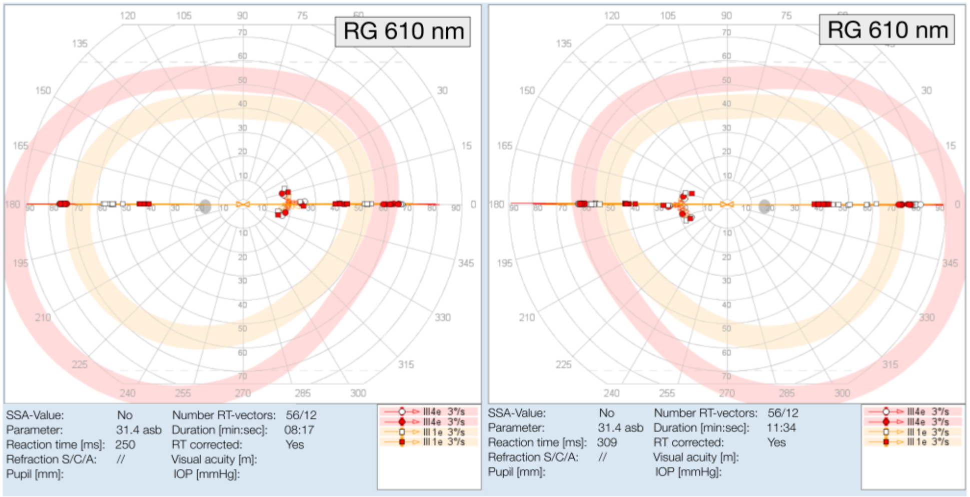

PRO-13, male, 26y. (2020-01-07)

LE **Normal trichromasia** RE

J

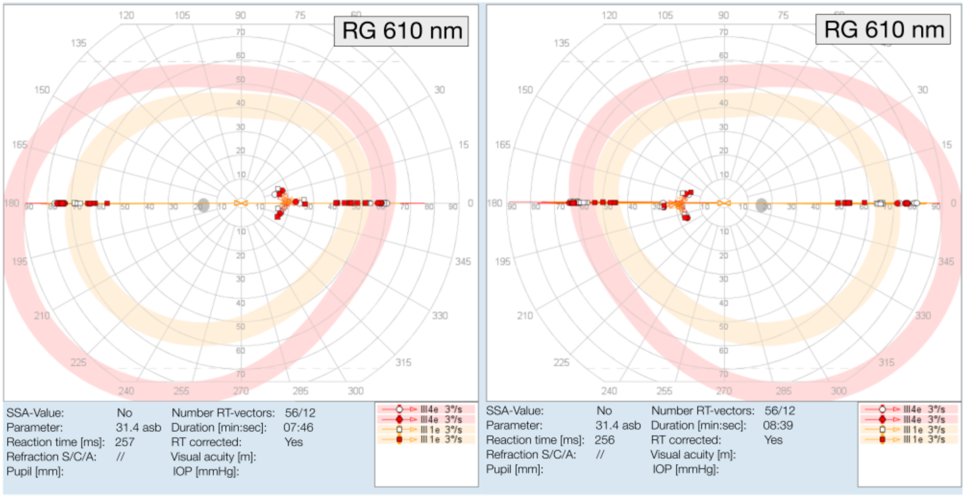

PRO-14, male, 24y. (2020-01-13)

LE **Normal trichromasia** RE

K

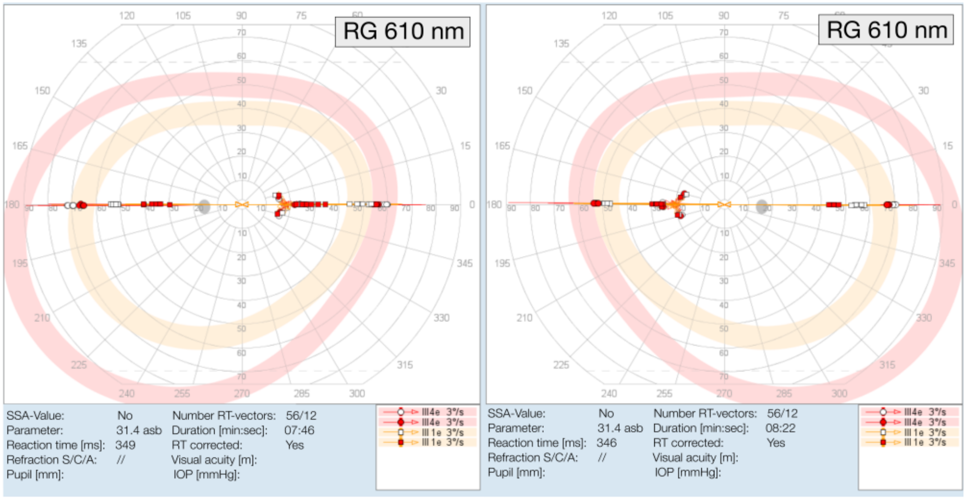

PRO-15, male, 49y. (2020-01-13)
