# Supplementary material for: Dissociation between red and white stimulus perception: A perimetric quantification of protanopic color vision deficiencies
Source: PLoS One. 2021 Dec 20;16(12):e0260362. doi: 10.1371/journal.pone.0260362 (PMC8687589; doi:10.1371/journal.pone.0260362)
Supplement: S2 Fig — RWR analysis of the protanope subjects (A-E) and the normal trichromate PRO-11 (C), separately for the right (RE) and left eye (LE). The data points were drawn onto a visual field map, showing the locus of the subject’s stimuli detection. For analytical purposes the points are presented as boxplots, visualizing the statistical parameters median (middle line) and the interquartile range (IQR: 25th and 75th quartile, lateral borders of the box). By connecting the corresponding median thresholds of each condition the horizontal extent (horizontal lines) was obtained for the GOLDMANN stimulus characteristics III4e (thick lines) and III1e (thin lines). The RWR was calculated by dividing the III4e red extent by the III4e white extent, III1e respectively. (PDF) [file pone.0260362.s002.pdf]

Supplemental Digital Content 6: RWR-figures (figure description at the end)

A

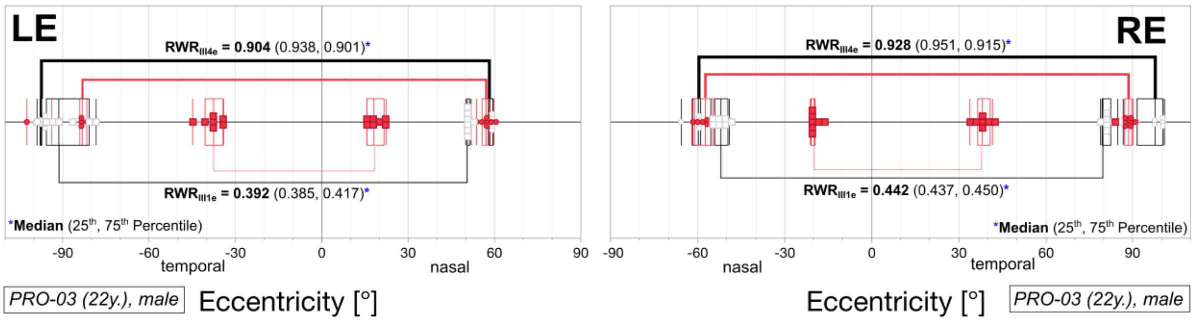

B

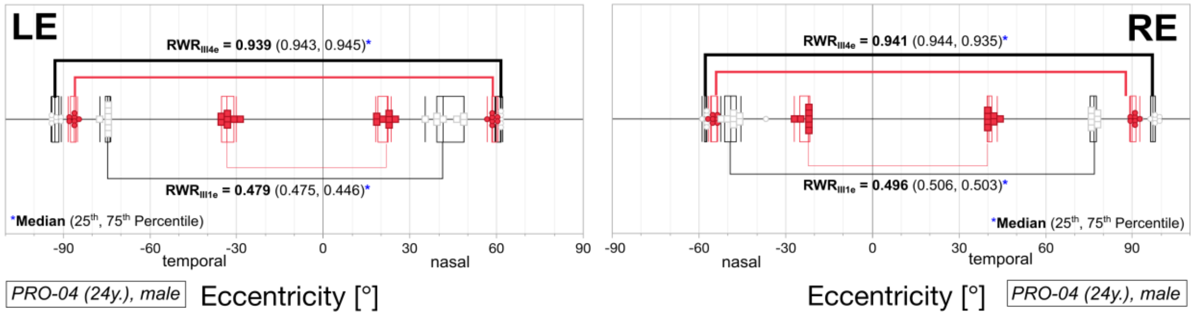

C

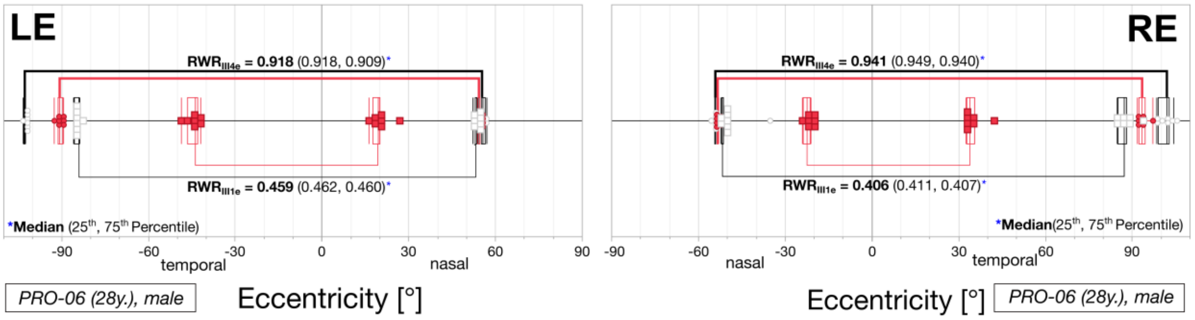

D

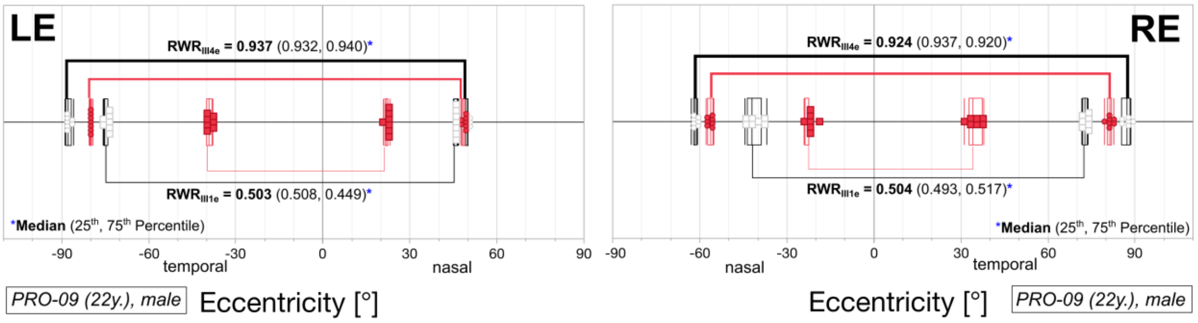

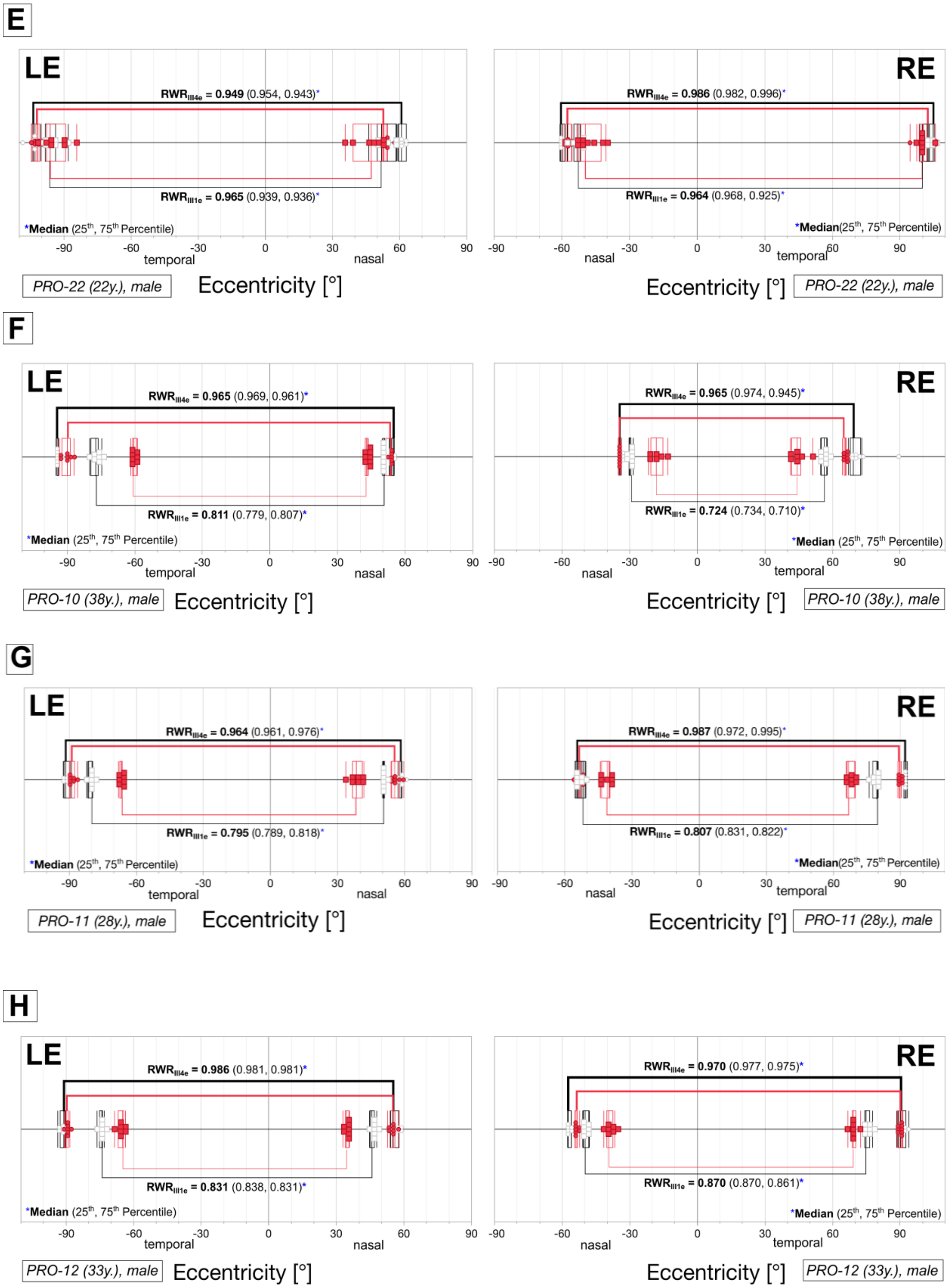
E
LE
RWR_{III4e} = 0.949 (0.954, 0.943)*
RWR_{III1e} = 0.965 (0.939, 0.936)*
*Median (25th, 75th Percentile)
-90 -60 -30 0 30 60 90
temporal
nasal
PRO-22 (22y.), male
Eccentricity [°]
RE
RWR_{III4e} = 0.986 (0.982, 0.996)*
RWR_{III1e} = 0.964 (0.968, 0.925)*
*Median(25th, 75th Percentile)
-90 -60 -30 0 30 60 90
nasal
temporal
Eccentricity [°]
PRO-22 (22y.), male
F
LE
RWR_{III4e} = 0.965 (0.969, 0.961)*
RWR_{III1e} = 0.811 (0.779, 0.807)*
*Median (25th, 75th Percentile)
-90 -60 -30 0 30 60 90
temporal
nasal
PRO-10 (38y.), male
Eccentricity [°]
RE
RWR_{III4e} = 0.965 (0.974, 0.945)*
RWR_{III1e} = 0.724 (0.734, 0.710)*
*Median (25th, 75th Percentile)
-90 -60 -30 0 30 60 90
nasal
temporal
Eccentricity [°]
PRO-10 (38y.), male
G
LE
RWR_{III4e} = 0.964 (0.961, 0.976)*
RWR_{III1e} = 0.795 (0.789, 0.818)*
*Median (25th, 75th Percentile)
-90 -60 -30 0 30 60 90
temporal
nasal
PRO-11 (28y.), male
Eccentricity [°]
RE
RWR_{III4e} = 0.987 (0.972, 0.995)*
RWR_{III1e} = 0.807 (0.831, 0.822)*
*Median(25th, 75th Percentile)
-90 -60 -30 0 30 60 90
nasal
temporal
Eccentricity [°]
PRO-11 (28y.), male
H
LE
RWR_{III4e} = 0.986 (0.981, 0.981)*
RWR_{III1e} = 0.831 (0.838, 0.831)*
*Median (25th, 75th Percentile)
-90 -60 -30 0 30 60 90
temporal
nasal
PRO-12 (33y.), male
Eccentricity [°]
RE
RWR_{III4e} = 0.970 (0.977, 0.975)*
RWR_{III1e} = 0.870 (0.870, 0.861)*
*Median (25th, 75th Percentile)
-90 -60 -30 0 30 60 90
nasal
temporal
Eccentricity [°]
PRO-12 (33y.), male

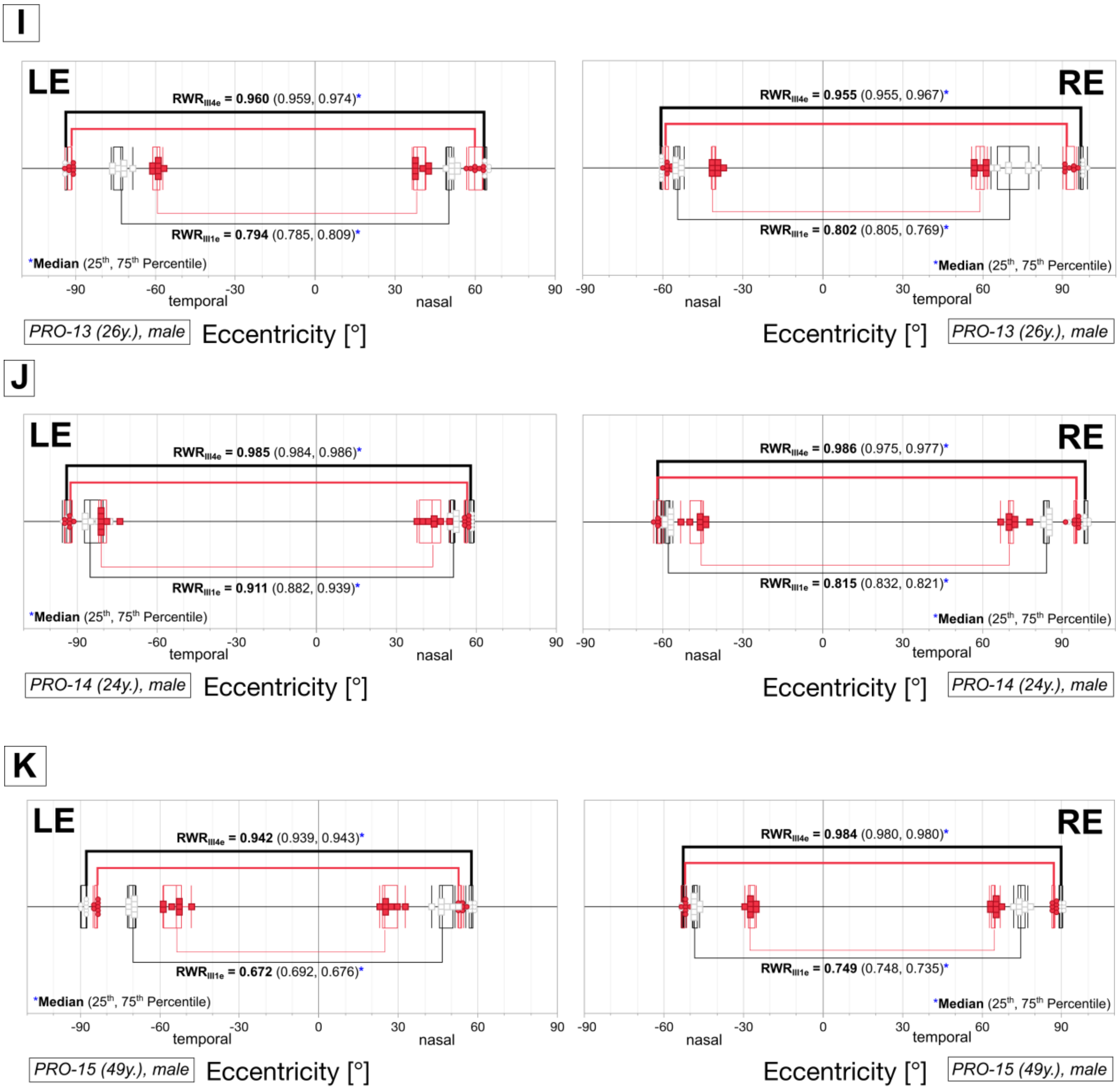

Figure: RWR analysis of the protanope subjects (A-E) and the normal trichromate PRO-11 (C), separately for the right (RE) and left eye (LE). The data points were drawn onto a visual field map, showing the locus of the subject's stimuli detection. For analytical purposes the points are presented as boxplots, visualizing the statistical parameters *median* (middle line) and the *interquartile range* (IQR: 25th and 75th quartile, lateral borders of the box). By connecting the corresponding median thresholds of each condition the horizontal extent (horizontal lines) was obtained for the GOLDMANN stimulus characteristics III4e (thick lines) and III1e (thin lines). The RWR was calculated by dividing the III4e red extent by the III4e white extent, III1e respectively
