# Supplementary material for: Dissociation between red and white stimulus perception: A perimetric quantification of protanopic color vision deficiencies
Source: PLoS One. 2021 Dec 20;16(12):e0260362. doi: 10.1371/journal.pone.0260362 (PMC8687589; doi:10.1371/journal.pone.0260362)
Supplement: S1 Table — Medical record sheet for the documentation of the subject’s general and ophthalmological optical history. (PDF) [file pone.0260362.s003.pdf]

Supplemental Digital Content 1: medical records sheet

Medical record sheet for the documentation of the subject's general and ophthalmological optical history

**Augenanamnese**				
	**"j" für ja und "n" für nein**	**Welches Auge ist betroffen? "R" für rechts, "L" für links**	**Falls ja, in welchem Lebensjahr Erstdiagnose/ Erstversorgung?**	**Erläuterungen / Ergänzungen**
Brillenträger?				
Kontaktlinsenträger?				
Sehen Sie auf beiden Augen mit Korr. gleich gut?				
Ist eine Schwach-sichtigkeit bekannt? (Amblyopie)				
Schielen bekannt?				
Augenbewegungsstörungen bzw. Doppelbilder?				
Grauer Star (Katarakt)?				
Grüner Star (Glaukom)?				
Netzhauterkrankungen? (z.B. Netzhautablösung, …)				
Erkrankungen an der Stelle des schärfsten Sehens (Makula)?				
Schwere chronische Augenentzündungen?				
Augenverletzungen?				
Augen-Operationen?				

**Familienanamnese**		
	**"j" für ja und "n" für nein**	**Erläuterungen / Ergänzungen**
Hohe Fehlsichtigkeiten?		
Glaukom (Grüner Star)?		
Katarakt (Grauer Star)?		
Netzhautablösung?		
Farbsehstörungen?		
Augenmedikamente (Tropfen/Salben)?		

**Allgemeinanamnese**		
	**"j" für ja und "n" für nein**	**Erläuterungen / Ergänzungen**
Körpergröße [m]		
Körpermasse [kg]		
Rechtshänder		
Linkshänder		
Beidhändig		
Verwechseln sie häufig rechts und links?		
Krankheiten, die den Nachtschlaf beeinträchtigen? (Schlafapnoe, Restless Legs, andere Schlafstörungen)		
Anderweitige Schlafstörungen?		
Zu welcher Uhrzeit...<br><br>gehen Sie gewöhnlich ins Bett?<br><br>stehen Sie normalerweise auf?		

Allgemeine Erkrankungen			
	**"j" für ja und "n" für nein**	**Falls ja, ab welchem Lebensjahr / wann wurde Erstdiagnose gestellt?**	**Erläuterungen / Ergänzungen**
Herz-Kreislauferkrankung?			
Herzrhythmusstörungen			
Herzinfarkt / bekannte Verengung der Herzkranzgefäße			
Bluthochdruck			
Haben Sie einen Herz-schrittmacher oder andere elektronisch aktive Implantate?			
Andere Organerkrankung? (z.B. Lunge, Leber, Niere, Magen, Darm…)			
Neurologische Erkrankungen? (z.B. Schlaganfall, Epilepsie,…)			
Stoffwechselerkrankung?			
Blutzuckererkrankung = Diabetes mellitus			
Schilddrüsen-*Über*funktion (= *Hyper*thyeose)			
Schilddrüsen-*Unter*funktion (= *Hypo*thyreose)			
Fettstoffwechsel			
Sonstige			
Schwangerschaft?			
Seelische Erkrankungen?			
Andere Erkrankungen?			

	"j" für ja und "n" für nein	Falls ja, ab welchem Lebensjahr / wann wurde Erstdiagnose gestellt?	Erläuterungen / Ergänzungen
Nikotinkonsum			
Alkoholkonsum			
Medikamenteneinnahme?			
Dauermedikation? (d.h. zumindest seit 6 Monaten)			
Medikamente, welche die Reaktionszeit beeinflussen? (Bsp.: Antiepileptika, Antihistaminika, Antidepressiva, etc.)			
Operationen			
**Haben Sie Allergien?**			
Hinweis:		***Keine Gabe von Neosynephrin***	

**Sozialanamnese**		
Beruf		
Muttersprache		
Höchster Bildungsabschluss (entsprechend 7 ISCED Bildungskategorien)		Hinweis:<br>1 Grundschule; 2 Hauptschul-/Realschulabschluss/Gymnasium (bis Klasse 10); 3 gymnasiale Oberstufe/Fachgymnasium; 4 Berufliche Ausbildung/Berufsfachschule; 5 Fachhochschulreife; 6 Bachelor oder Vergleichbares; 7 Master oder Vergleichbares; 8 Doktorgrad oder Vergleichbares

**Fahrbezogene Fragen**				
	**"j" für ja und "n" für nein**	**Falls ja, ab welchem Lebensjahr?**	**Klasse**	**Erläuterungen / Ergänzungen**
Führerschein				
Wie oft sind Sie mit dem PKW unterwegs? (Ankreuzen)				
- weniger als 1 Mal pro Woche				
- 1 bis 2 Mal pro Woche				
- 3 bis 5 Mal pro Woche				
- (fast) täglich				
Jährliche Fahrstrecke [km]				
**Wie groß ist der prozentuale Anteil an:**				
- Stadtfahrten				
- Landstraßenfahrten				
- Autobahnfahrten				
Was für ein Auto fahren Sie derzeit?				
Unfälle		**Art des Unfalls (Bsp.: Vorfahrt, Auffahrunfall, Anzahl, zu welcher Tageszeit, etc.):**		
Haben Sie bereits Erfahrung mit Fahrerassistenzsystemen (z.B. Navigationssystem, Tempomat etc.)?				
Haben Sie Erfahrung mit Smartphones und/oder Tablets?				

**Persönliche Selbsteinschätzung**		
	**schlecht**	**gut**
Bitte markieren Sie Ihre persönliche Selbst-Einschätzung durch einen senkrechten Strich auf jeder der Linie		
Wie schätzen Sie ihr Farbsehvermögen ein?		
Wie schätzen Sie Ihre Blendungsempfindlichkeit ein?		
Wie schätzen Sie Ihre Nachtfahrtauglichkeit ein?		
Wie schätzen Sie Ihre Tagfahrtauglichkeit ein?		

**wird vom Personal ausgefüllt**	**"j" für ja und "n" für nein**
**Aufklärungsmaterial vorhanden?**	
**Datenschutzerklärung vorhanden?**	
**Fragebogen NEI VFQ-25 vorhanden?**	

Source: „PROLicht“ study documents
