# Supplementary material for: Dissociation between red and white stimulus perception: A perimetric quantification of protanopic color vision deficiencies
Source: PLoS One. 2021 Dec 20;16(12):e0260362. doi: 10.1371/journal.pone.0260362 (PMC8687589; doi:10.1371/journal.pone.0260362)
Supplement: S2 Table — Standard examination diagnosis sheet for the documentation of the subject’s performance or health findings. (PDF) [file pone.0260362.s004.pdf]

Supplemental Digital Content 2: standard examination diagnosis sheet

Standard examination diagnosis sheet for the documentation of the subject's performance or health findings

**Stereosehen (Lang-Stereotest-I (Fronhäuser, Prüfentfernung 40 cm))**			
**Objekt in Winkelsekunden ['']:**	**Erkannt**	**Nicht erkannt**	**Bemerkungen**
550 (Auto)			
600 (Stern)			
1200 (Katze)			
Stereosehen intakt/auffällig			

**Augenstellung und Motilität**					
	**RA Intakt/Unauffällig**	**RA Gestört/Auffällig**	**LA Intakt/Unauffällig**	**LA Gestört/Auffällig**	**Bemerkung-en**
Augenstellung<br>Einseitiger und alternierender Abdecktest bei Fixation einer Lichtquelle in Ferne und Nähe					
Augenbeweglichk eit					

Pupillen (Efferenz und Afferenz): Swinging Flashlighttest					
	RA		LA		
	intakt/ unauffällig	gestört/ auffällig	intakt/ unauffällig	gestört/ auffällig	Bemerkungen
**Pupillen**					
Pupillen rund					
Pupillen zentriert					
In Helligkeit und Dunkelheit isokor?					
**Anisokorie**	☐ **ja, dann weiter ausfüllen ↓** ☐ **nein, dann weiter mit RAPD** *(Bitte ankreuzen!)*				
R > L					
R < L					
Bei Dunkelheit zunehmend?					
Bei Helligkeit zunehmend?					
**RAPD?**		__ , __ logE	__ , __ logE		
Sonstige Anmerkungen					

**Vorderer Augenabschnitt**					Spaltlampe (BQ900, Haag Streit, Köniz)
	**RA Intakt/ Unauffällig**	**RA Gestört/ Auffällig**	**LA Intakt/ Unauffällig**	**LA Gestört/ Auffällig**	**Bemerkungen**
Lider					
Bindehaut					
Hornhaut					
Vorderkammer					
Vorderkammertiefe temporale Peripherie (Grad 1-4, nach W. van Herick)					
Irisfarbe					
Iris/Pupille					
Linse					
Glaskörper					
Intraokular reizfrei?					

**Augenhintergrund**<br>BETA 200 S LED Ophthalmoskop, Fa. Heine Optotechnik, Herrsching, OMEGA 500, Fa. Heine Optotechnik, Herrsching/D						
	**RA**				**LA**	
	**Intakt/ Unauffällig**	**Gestört/ Auffällig**	**Bemerkungen**	**Intakt/ Unauffällig**	**Gestört/ Auffällig**	**Bemerkungen**
Papille randscharf?						
Papille vital gefärbt?						
Zentrale Exkavation						
CDR:						
ISNT Regel erfüllt?						
Nervenfaser-schicht (rotfrei)						
	**Ausprägungs-grad**		**Bemerkungen**	**Ausprägungs-grad**		**Bemerkungen**
Wallreflex (+ / (+) / ø)						
Foveolarreflex (+ / (+) / ø)						
	**Intakt/ Unauffällig**	**Gestört/ Auffällig**	**Bemerkungen**	**Intakt/ Unauffällig**	**Gestört / Auffällig**	**Bemerkungen**
Zentrale Fixation						
Exzentrischer Fixationsort:						
Gefäße						
a : v =						
Peripherie						
**allgemeine Bemerkungen**						

Source: “PROLicht” study documents
