# Supplementary material for: Dissociation between red and white stimulus perception: A perimetric quantification of protanopic color vision deficiencies
Source: PLoS One. 2021 Dec 20;16(12):e0260362. doi: 10.1371/journal.pone.0260362 (PMC8687589; doi:10.1371/journal.pone.0260362)
Supplement: S3 Table — Subject’s (PRO-ID as identification) habitual correction, objective/subjective refraction (sphere/cylindre/and visual acuity (VAsc = visual acuity without correction; VAcc = visual acuity with correction) listed for the right eye (RE), the left eye (LE) and binocularly (bin.). (PDF) [file pone.0260362.s005.pdf]

Supplemental Digital Content 4: Subject's visual acuity and habitual correction

Subject's (PRO-ID as identification) habitual correction, objective/subjective refraction (sphere/cylindre/and visual acuity (VA_{sc} = visual acuity without correction; VA_{cc} = visual acuity with correction) listed for the right eye (RE), the left eye (LE) and binocularly (bin.)

PRO-ID	Color vision	Age [years]	Correction RE	Correction LE	Objective refraction RE	Objective refraction LE	Distance visual acuity VA_{sc} (RE/LE/bin.			Distance visual acuity VA_{cc} (RE/LE/bin.			Subjective refraction RE	Subjective refraction LE	Visual acuity after subj. refraction (RE/LE/bin.)		
**PRO-10**	**Normal trichromasia**	37,7	-1.50 -1.00 x 11	-1.50 -1.00 x 3	-2.50 -0.75 x 11	-1.75 -0.75 x 179	-	-	-	-	-	-	-	-	-	-	-
**PRO-11**	**Normal trichromasia**	28,4	-	-	+0.25 -0.25 x 120	+0.50 -0.25 x 45	2,00	1,60	2,00	-	-	-	-	-	-	-	-
**PRO-12**	**Normal trichromasia**	32,6	-0.50 -0.25 x 15	-0.5	+0.25 -0.25 x 10	+0.25 -0.25 x 0	1,00	2,00	2,00	1,60	2,00	2,00	0.00 -0.50 x 20	-	2,00	2,00	2,00
**PRO-13**	**Normal trichromasia**	26,0	-0.25 -0.25 x 109	-0.50 -0.50 x 156	+0.25 -0.25 x 130	+0.25 -0.25 x 7	1,60	1,60	2,00	2,00	2,00	2,50	-	-	-	-	-
**PRO-14**	**Normal trichromasia**	23,5	-	-	+1.00 -0.25 x 20	+0.75 -0.50 x 148	1,60	2,00	-	-	-	-	+0.75 -0.50 x 29	+0.25	2,00	2,00	2,00
**PRO-15**	**Normal trichromasia**	49,1	-	-	0.00 -0.25 x 176	-0.25	1,25	0,80	1,25	-	-	-	-0.25 -0.25 x 176	-0.50 -0.25 x 37	1,60	1,60	1,60
**PRO-03**	**Protanopia**	21,8	-	-	+0.50	+0.75 -0.25 x 178	1,60	1,60	1,60	-	-	-	-	+0.75	2,00	1,60	2,00
**PRO-04**	**Protanopia**	24,1	-	-	+0.75 -0.75 x 104	+0.50 -0.25 x 81	2,00	2,00	2,50	-	-	-	+0.75 -0.75 x 80	+0.50 -0.25 x 120	1,60	2,00	2,00
**PRO-06**	**Protanopia**	28,6	-	-	+2.50 -0.75 x 107	+1.75 -1.00 x 74	1,25	1,25	1,60	-	-	-	+2.25 -1.25 96	+1.00 -1.50 x 90	1,25	1,25	1,60
**PRO-09**	**Protanopia**	22,1	-	-	+0.25 -1.00 x 13	-	0,80	0,80	1,00	-	-	-	-0.25 -1.00 x 18	-0.75 -0.25 x 170	1,25	1,25	1,25
**PRO-22**	**Protanopia**	22,0	-0.25 -0.75 x 27	-0.25 -0.75 x 170	+0.50 -0.75 x 25	+0.50 -0.75 x 172	1,00	1,25	1,25	1,60	1,60	1,60	-0.25 -0.75 x 32	-0.50 -0.50 x 173	1,60	1,60	2,00
