# Supplementary material for: Dissociation between red and white stimulus perception: A perimetric quantification of protanopic color vision deficiencies
Source: PLoS One. 2021 Dec 20;16(12):e0260362. doi: 10.1371/journal.pone.0260362 (PMC8687589; doi:10.1371/journal.pone.0260362)
Supplement: S4 Table — Red/white dissociation ratio results for every participant (PRO-ID as identification for the right eye (RE), left eye (LE) and both eyes (BE), seperately for the intensities III4e and III1e (shaded grey and bold letters). (PDF) [file pone.0260362.s006.pdf]

Supplemental Digital Content 7: complete data table of the RWR median

Red/white dissociation ratio results for every participant (PRO-ID as identification for the right eye (RE), left eye (LE) and both eyes (BE), seperately for the intensities III4e and III1e (shaded grey and bold letters)

			RE		LE		BE	
			RWR_{III4e}	RWR_{III1e}	RWR_{III4e}	RWR_{III1e}	RWR_{III4e}	RWR_{III1e}
PRO-ID	Color vision	Age [years]	Median	Median	Median	Median	Median	Median
Normal trichchromasia								
PRO-10	Normal trichromasia	37,7	0,965	**0,724**	0,965	**0,811**	0,972	**0,794**
PRO-11	Normal trichromasia	28,4	0,987	**0,807**	0,964	**0,795**	0,978	**0,785**
PRO-12	Normal trichromasia	32,6	0,970	**0,870**	0,986	**0,831**	0,962	**0,816**
PRO-13	Normal trichromasia	26,0	0,955	**0,802**	0,960	**0,794**	0,951	**0,702**
PRO-14	Normal trichromasia	23,5	0,986	**0,815**	0,985	**0,911**	0,988	**0,966**
PRO-15	Normal trichromasia	49,1	0,984	**0,749**	0,942	**0,672**	0,968	**0,793**
Group median			*0,977*	*0,805*	*0,965*	*0,803*	*0,970*	*0,794*
Group IQR *			*0,019*	*0,051*	*0,019*	*0,032*	*0,013*	*0,024*
Protanopia								
PRO-03	Protanopia	21,8	0,928	**0,442**	0,904	**0,392**	0,868	**0,388**
PRO-04	Protanopia	24,1	0,941	**0,496**	0,939	**0,479**	0,913	**0,303**
PRO-06	Protanopia	28,6	0,941	**0,406**	0,918	**0,459**	0,861	**0,399**
PRO-09	Protanopia	22,1	0,924	**0,504**	0,937	**0,503**	0,891	**0,383**
PRO-22	Protanopia	22,0	0,986	**0,964**	0,949	**0,965**	0,891	**0,874**
Group median			*0,941*	*0,496*	*0,937*	*0,479*	*0,891*	*0,388*
Group IQR *			*0,013*	*0,062*	*0,021*	*0,044*	*0,023*	*0,016*

** IQR= interquartile range
