# Supplementary material for: Dissociation between red and white stimulus perception: A perimetric quantification of protanopic color vision deficiencies
Source: PLoS One. 2021 Dec 20;16(12):e0260362. doi: 10.1371/journal.pone.0260362 (PMC8687589; doi:10.1371/journal.pone.0260362)
Supplement: S5 Table — Reaction times [sec] for all subjects (PRO-ID as identification) listed for the right eye (RE), left eye (LE) and both eyes (BE), separately for the intensities III4e and III1e (shaded grey and bold letters), for white and red respectively. The highlighted cells mark the false positive answers under the normal reference value of 180 ms (table extents over several pages). (PDF) [file pone.0260362.s007.pdf]

Supplemental Digital Content 8: complete data table of the reaction time assessment

Reaction times [sec] for all subjects (PRO-ID as identification) listed for the right eye (RE), left eye (LE) and both eyes (BE), separately for the intensities III4e and III1e (shaded grey and bold letters), for white and red respectively. The highlighted cells mark the false positive answers under the normal reference value of 180 ms (table extents over several pages)

			RE				LE				BE			
			RT_{III4e}		RT_{III1e}		RT_{III4e}		RT_{III1e}		RT_{III4e}		RT_{III1e}	
**PRO-ID**	**Color vision**	**Age [years]**	**RT*$_{Med}$ white**	**RT*$_{Med}$ red**	**RT*$_{Med}$ white**	**RT*$_{Med}$ red**	**RT*$_{Med}$ white**	**RT*$_{Med}$ red**	**RT*$_{Med}$ white**	**RT*$_{Med}$ red**	**RT*$_{Med}$ white**	**RT*$_{Med}$ red**	**RT*$_{Med}$ white**	**RT*$_{Med}$ red**
**Normal trichchromasia**														
**PRO-10**	**Normal trichromasia**	37,7	0,474	0,564	**0,505**	**0,472**	0,538	0,544	**0,593**	**0,550**	0,504	0,424	**0,593**	**0,583**
**PRO-11**	**Normal trichromasia**	28,4	0,447	0,458	**0,618**	**0,656**	0,544	0,451	**0,610**	**0,562**	0,398	0,487	**0,683**	**0,574**
**PRO-12**	**Normal trichromasia**	32,6	0,623	0,566	**0,475**	**0,590**	0,605	0,468	**0,647**	**0,669**	0,480	0,358	**0,671**	**0,576**
**PRO-13**	**Normal trichromasia**	26,0	0,586	0,375	**0,495**	**0,699**	0,582	0,489	**0,268**	**0,720**	1,104	0,616	**0,558**	**0,639**
**PRO-14**	**Normal trichromasia**	23,5	0,630	0,368	**0,455**	**0,530**	0,574	0,411	**0,635**	**0,734**	0,397	0,479	**0,438**	**0,500**
**PRO-15**	**Normal trichromasia**	49,1	0,765	0,588	**0,589**	**0,748**	0,840	0,748	**0,777**	**0,701**	0,855	0,694	**0,726**	**0,880**
Group median			***0,605***	***0,511***	***0,500***	***0,623***	***0,578***	***0,479***	***0,623***	***0,685***	***0,492***	***0,483***	***0,632***	***0,580***
Group IQR **			***0,126***	***0,170***	***0,088***	***0,143***	***0,048***	***0,075***	***0,047***	***0,127***	***0,349***	***0,146***	***0,113***	***0,051***
**Protanopia**														
**PRO-03**	**Protanopia**	21,8	0,468	0,548	**0,726**	**1,259**	0,816	0,458	**0,754**	**0,974**	0,474	0,621	**0,582**	**1,295**
**PRO-04**	**Protanopia**	24,1	0,545	0,504	**0,569**	**0,814**	0,609	0,419	**0,389**	**0,931**	0,628	0,589	**0,627**	**0,502**
**PRO-06**	**Protanopia**	28,6	0,724	0,614	**0,471**	**1,409**	0,549	0,503	**0,490**	**1,299**	0,497	0,670	**0,543**	**0,826**
**PRO-09**	**Protanopia**	22,1	0,554	0,632	**0,642**	**1,299**	0,686	0,773	**0,553**	**0,951**	0,619	0,531	**0,436**	**0,717**
**PRO-22**	**Protanopia**	22,0	0,430	0,471	**0,281**	**1,260**	0,538	0,560	**0,530**	**0,895**	0,449	0,442	**0,457**	**0,679**
Group median			***0,545***	***0,548***	***0,569***	***1,260***	***0,609***	***0,503***	***0,530***	***0,951***	***0,497***	***0,589***	***0,543***	***0,717***
Group IQR **			***0,086***	***0,110***	***0,171***	***0,040***	***0,137***	***0,102***	***0,063***	***0,043***	***0,145***	***0,090***	***0,125***	***0,147***

*RT= reaction time [s]

**IQR= interquartile range
