# Supplementary material for: Dissociation between red and white stimulus perception: A perimetric quantification of protanopic color vision deficiencies
Source: PLoS One. 2021 Dec 20;16(12):e0260362. doi: 10.1371/journal.pone.0260362 (PMC8687589; doi:10.1371/journal.pone.0260362)
Supplement: S6 Table — NEI-VFQ-scoring for every participant (PRO-ID as identification) represented by the different thematic parts of the questionnaire, highlighted is the color vision score for comparison purpose between protanopia and normal trichromasia. (PDF) [file pone.0260362.s008.pdf]

**Supplemental Digital Content 9: table with the resulting scores of the NEI-VFQ questionnaire for each category**

NEI-VFQ-scoring for every participant (PRO-ID as identification) represented by the different thematic parts of the questionnaire, highlighted is the color vision score for comparison purpose between protanopia and normal trichromasia

| PRO-ID | Color Vision | General Health | General Vision | Ocular pain | Near Vision | Distance Vision | Social Functioning | Mental Health | Role Functioning | Dependency due to vision | Driving | Color vision | Peripheral vision | Total score |
|---|---|---|---|---|---|---|---|---|---|---|---|---|---|---|
| **PRO-10** | **Normal trichromasia** | 60 | 70 | 87,5 | 100 | 91,7 | 100 | 95 | 43,8 | 100 | 83,3 | 100 | 100 | 1031,3 |
| **PRO-11** | **Normal trichromasia** | 87,5 | 100 | 87,5 | 100 | 100 | 100 | 100 | 100 | 100 | 100 | 100 | 100 | 1175,0 |
| **PRO-12** | **Normal trichromasia** | 77,5 | 90 | 87,5 | 95,8 | 88 | 83,3 | 90 | 75 | 100 | 75 | 100 | 75 | 1036,6 |
| **PRO-13** | **Normal trichromasia** | 95 | 90 | 100 | 100 | 100,0 | 100 | 100 | 93,8 | 100 | 100 | 100 | 100 | 1178,8 |
| **PRO-14** | **Normal trichromasia** | 82,5 | 80 | 87,5 | 95,8 | 100,0 | 100 | 95 | 68,8 | 100 | 91,7 | 100 | 100 | 1101,3 |
| **PRO-15** | **Normal trichromasia** | 65 | 80 | 87,5 | 87,5 | 95,8 | 100 | 90 | 81,3 | 100 | 83,3 | 100 | 100 | 1070,4 |
| **Group median** | | **80** | **85** | **87,5** | **97,9** | **97,9** | **100** | **95** | **78,2** | **100** | **87,5** | **100** | **100** | **1085,9** |
| **Group IQR*** | | **18,1** | **10,0** | **0,0** | **4,2** | **7,3** | **0,0** | **7,5** | **20,3** | **0,0** | **14,6** | **0,0** | **0,0** | **111,5** |
| | | | | | | | | | | | | | | |
| **PRO-03** | **Protanopia** | 77,5 | 90 | 100 | 100 | 95,8 | 100 | 100 | 87,5 | 100 | 100 | N/A | 100 | 1050,8 |
| **PRO-04** | **Protanopia** | 100 | 90 | 87,5 | 100 | 100,0 | 100 | 90 | 75 | 75 | 100 | 25 | 100 | 1042,5 |
| **PRO-06** | **Protanopia** | 77,5 | 90 | 87,5 | 100 | 95,8 | 100 | 95 | 87,5 | 100 | 100 | 100 | 100 | 1133,3 |
| **PRO-09** | **Protanopia** | 77,5 | 75 | 100 | 100 | 95,8 | 100 | 95 | 75 | 100 | 100 | 100 | 100 | 1118,3 |
| **PRO-22** | **Protanopia** | 60 | 85 | 100 | 100 | 95,8 | 100 | 100 | 93,8 | 100 | 83,3 | 75 | 100 | 1092,9 |
| **Group median** | | **77,5** | **90** | **100** | **100** | **95,8** | **100** | **95** | **87,5** | **100** | **100** | **87,5** | **100** | **1092,9** |
| **Group IQR*** | | **0,0** | **5,0** | **12,5** | **0,0** | **0,0** | **0,0** | **5,0** | **12,5** | **0,0** | **0,0** | **37,5** | **0,0** | **67,5** |

*IQR = interquartile range
