# Supplementary material for: Dissociation between red and white stimulus perception: A perimetric quantification of protanopic color vision deficiencies
Source: PLoS One. 2021 Dec 20;16(12):e0260362. doi: 10.1371/journal.pone.0260362 (PMC8687589; doi:10.1371/journal.pone.0260362)
Supplement: S1 File — Detailed description of the experimental setup and the protocol of the spectroradiophotometric measurements. (PDF) [file pone.0260362.s009.pdf]

Supplemental Digital Content 3: detailed description of the experimental setup and the protocol of the spectroradiophotometric measurements

Experimental set up:

Spectroradiophotometric measurements (spectroradiometer CAS140 CT VIS/UV (Instrument Systems, Munich/FRG)) were conducted by Prof. Dr. rer. J. Nolting (project leader of the spectroradiophotometric measurements) and Prof. Dr. G. Dittmar (Aalen/FRG), using the sensor head TOP100 with a measuring aperture of 0.25 mm and a measuring objective f = 60 mm, f/2.8. Technical consulting and development was provided by the Steinbeis Transfer Center (Karlsruhe/FRG). SpecWin Pro 3.3 (Instrument Systems, Munich/FRG) was used as measurement software.

The measurements were examined in the center of the perimetric cupola for the expert-opinion relevant stimulus III4e and the dimmer stimulus III1e. The following luminance were obtained (luminance data for the red stimulus with otherwise identical photometric Goldmann specifications are shown in brackets):

III4e: 306.9 cd/m^2, minus the “additionally measured” background luminance of 10 cd/m^2, resulting in: 296.9 cd/m^2 (104.3 cd/m^2, minus the background luminance: 94.3 cd/m^2, resulting in an „attenuation factor“ of 3.1, according to 5.0 dB)

III1e: 20.2 cd/m^2, minus the background luminance: 10.2 cd/m^2 (12.8 cd/m^2, minus the above-mentioned background luminance: 2.8 cd/m^2, resulting attenuation factor 3.6, according to 5.6 dB).

Projekt BASt ProLicht
Photometrische Vermessung von Fixationsmarken eines Perimeters

Messungen durchgeführt durch Jürgen Nolting
Mitarbeit: Schiefer, Ungewiß

Datum: 11.2.2020

Messgerät:
Spektroradiometer CAS 140 VIS/UV
Messkopf TOP100 mit Objektiv f=60mm, f/2,8
Minolta Luminance Meter LS160

Messdistanz: 45,7cm

Messobjekt: Perimeter Octopus 900 RWR mit zusätzlichen Projektionslasern

Leuchdichten der Fixationsmarken

Fixationsmarke	Leuchtdichte cd/m²	Farbe	Kurve
zentral	33,7	grün	1
Fixationslaser rechts	103,3	grün	2
Fixationslaser links	160,9	grün	3
Marke III 4 E zentral	306,9	weiß, 7602K	4
Marke V 4 E zentral	317,9	weiß, 7995K	5
Marke III 3 E zentral	103,9	weiß, 7214K	6
Marke III 2 E zentral	39,4	weiß, 6431K	7
Marke III 1 E zentral	20,2	weiß, 5472K	8
Marke III 4 E rot zentral	104,3	rot	9
Marke III 3 E rot zentral	39,7	rot	10
Marke III 2 E rot zentral	19,1	rot	11
Marke III 1 E rot zentral	12,8	rot	12
Marke III 4 E rot 40° rechts	97,8	rot	13
Marke III 4 E rot 30° links	98,5	rot	14
Vergleichsmessung mit Minolta Luminance Meter			
Marke V 4 E zentral	320,1		
Marke III 4 E	54,0		

Die erste Vergleichsmessung ist in guter Übereinstimmung mit der Messung zu Kurve 5. Die zweite Vergleichsmessung (III 4 E) weicht deutlich ab. Leider wurde nicht dokumentiert, ob die rote oder weiße Marke vermessen wurde (vgl. Kurve 4 oder 9). Bei Vermessung der roten Marke ist bei Messung mit dem Luminance Meter zwar mit größeren Abweichungen zu rechnen, die Größe der hier gefundenen Abweichung ist aber ungewöhnlich. Wahrscheinlich liegt ein Messfehler vor.

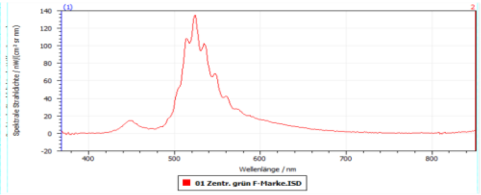

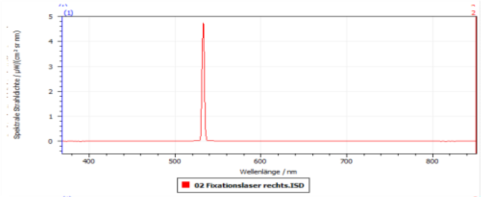

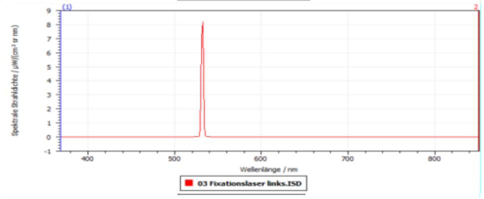

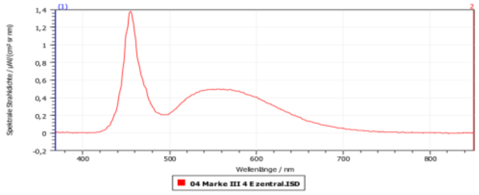

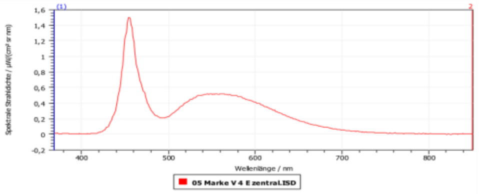

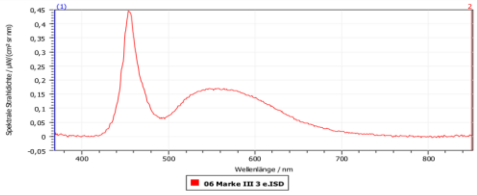

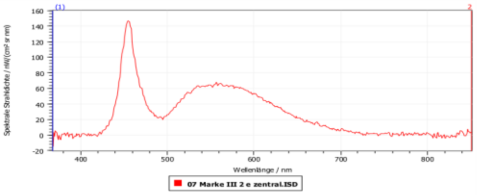

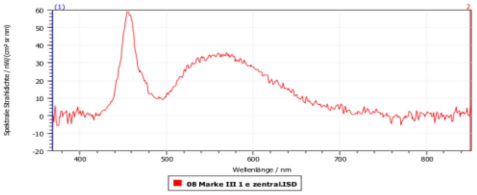

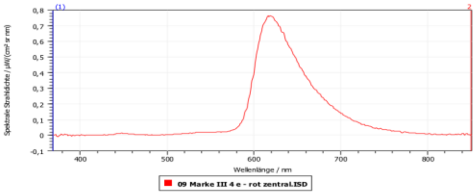

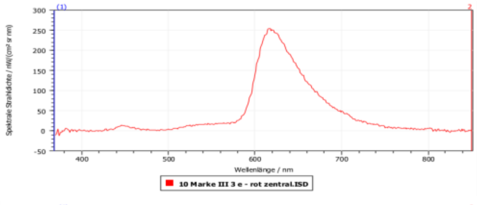

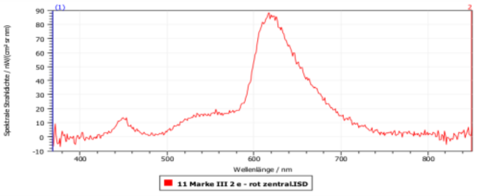

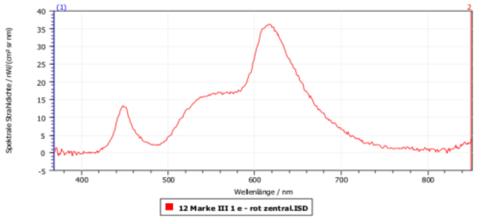

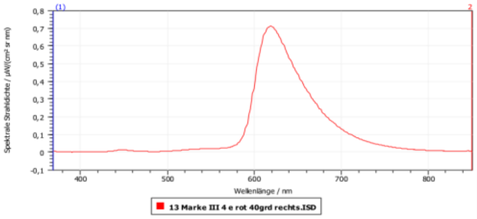

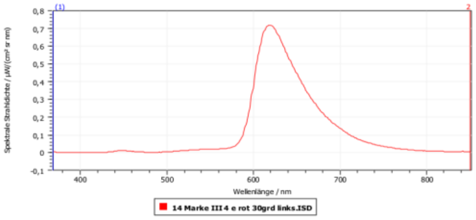

Aalen, 18.2.2020

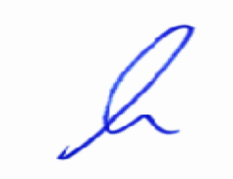

Source: “PROLicht“ study documents (conducted by the Competence Center Vision Research, Aalen (FRG)), issued by Prof. Dr. rer. J. Nolting in collaboration of Prof. Dr. med. U. Schiefer
